# Small-Molecule Inhibitors of GSK-3: Structural Insights and Their Application to Alzheimer's Disease Models

**DOI:** 10.1155/2012/381029

**Published:** 2012-07-22

**Authors:** Thomas Kramer, Boris Schmidt, Fabio Lo Monte

**Affiliations:** Clemens Schöpf-Institute of Organic Chemistry and Biochemistry, Technische Universität Darmstadt, 64287 Darmstadt, Germany

## Abstract

The world health organization (WHO) estimated that 18 million people are struck by Alzheimer's disease (AD). The USA, France, Germany, and other countries launched major programmes targeting the identification of risk factors, the improvement of caretaking, and fundamental research aiming to postpone the onset of AD. The glycogen synthase kinase 3 (GSK-3) is implicated in multiple cellular processes and has been linked to the pathogenesis of several diseases including diabetes mellitus, cancer, and AD. Inhibition of GSK-3 leads to neuroprotective effects, decreased **β**-amyloid production, and a reduction in tau hyperphosphorylation, which are all associated with AD. Various classes of small molecule GSK-3 inhibitors have been published in patents and original publications. Herein, we present a comprehensive summary of small molecules reported to interact with GSK-3. We illustrate the interactions of the inhibitors with the active site. Furthermore, we refer to the biological characterisation in terms of activity and selectivity for GSK-3, elucidate *in vivo* studies and pre-/clinical trials.

## 1. Introduction

Protein kinases regulate diverse cellular functions and thus are frequently exploited in drug discovery programmes [1]. They regulate signal transduction processes by phosphorylating serine, threonine and tyrosine residues in key proteins. The signalling pathways involved contribute to the pathology in many diseases [2, 3]. Glycogen synthase kinase 3 (GSK-3) was identified in the late 1970s and is a constitutively active, ubiquitous expressed serine/threonine kinase, which participates in a number of physiological processes ranging from glycogen metabolism to gene transcription [4]. Initially, the focus of pharmaceutical companies concerning GSK-3 was on diabetes mellitus, but since GSK-3 was linked to Alzheimer's disease (AD), the focus has moved from diabetes to AD. GSK-3 has been linked to all primary abnormalities associated with AD. GSK-3 interacts with different components of the plaque producing amyloid system, participates in phosphorylating the microtubule binding protein tau that contributes to the formation of neurofibrillary tangles, and has an influence on presenilin and other AD-associated proteins [4–8]. Two related isoforms of GSK-3 are present in mammalians, GSK-3*α* and *β*, which share 98% homology in their catalytic domains and have similar biochemical properties [9]. The isoforms are similar in their catalytic domains, yet differ significantly in their N-terminal regions [10]. Alois Alzheimer's first report of the neuropathological hallmarks of AD dates back to 1907 [11, 12]. The histopathology of the AD brain is characterized by the presence of abnormal filamentous tau-protein inclusions in nerve cells and extracellular amyloid deposits [13, 14]. Partially phosphorylated tau contains sequence motifs that support association with tubulin, which entails the stabilization of microtubules in AD-uneffected cells. The pathological hyperphosphorylation of these motifs destabilizes microtubules and consequently interferes with tubulin binding. The misfolding of hyperphosphorylated tau involves the E/Z-isomerisation of a phosphorylated Ser-Pro motive, which leads to the formation of insoluble neurofibrillary tangles (NFTs) and intraneuronal aggregates of paired helical filaments (PHFs) [9, 15]. GSK-3 phosphorylates multiple sites on tau both *in vitro* and *in vivo *[9]. It exerts a central and crucial role in the pathogenesis of both familial and sporadic forms of AD. Early-onset forms of familial Alzheimer's disease (FAD) have been linked to mutations in amyloid precursor protein (APP), presenilin-1 (PS-1), and presenilin-2 (PS-2). These mutations adversely affect APP processing and result in the increased production of the 40–42 amino acid long *β*-Amyloid (A*β*) peptides, which are the major component of amyloid deposits. Several risk factors have been associated with sporadic Alzheimer's disease (SAD). The most prevalent factors are aging and the presence of specific ApoE isoforms, which have been implicated in A*β* clearance. Sporadic Alzheimer's disease can be caused by the activation of *β*-secretase, which results in enhanced formation of A*β*. Enhanced A*β* production or deficiency in A*β* clearance will result in the deposition of A*β* aggregates [4, 16]. Recent work suggests that enhanced GSK-3 activity increases A*β* production [17]. Several studies support that GSK-3 inhibition leads to decreased A*β* production and a reduction in tau hyperphosphorylation [1].

A plethora of GSK-3 inhibitors has been described, and most of the biological effects were reported for *in vitro* and cellular studies [17]. These studies, the number of patent applications, and a successful phase II trial indicate that GSK-3 is a promising drug target for AD therapy, but the ultimate proof of concept has not been presented yet. GSK-3 is highly enriched in the brain, and several publications indicate that the GSK-3*β* isoform is a key kinase required for abnormal hyperphosphorylation of tau [18, 19]. Spittaels et al. generated a double-transgenic mouse overexpressing human protein tau and constitutively active human GSK-3*β* and ascertained that this kinase is implicated in aberrant tau phosphorylation and in addition reduced tau binding capacity to microtubules [15, 20]. The homology of the ATP-binding pocket in GSK-3*α* and GSK-3*β* presents an obstacle for the development of isoform selective inhibitors. All GSK-3 inhibitors developed until now are able to inhibit the two isoforms with similar potency, except **Λ–OS1 (36)**, which showed a selectivity (up to 7 fold) for GSK-3*α* [8, 21, 22]. 

The structures of GSK-3*β* cocrystallized with several inhibitors have been solved by X-ray crystallography recently. These structures provide a remarkable possibility to design both novel and selective GSK-3 inhibitors. There are two fundamental options to inhibit GSK-3: non-ATP competitive inhibition and ATP competitive inhibition.

The non-ATP competitive inhibitors, for example, substrate competitive inhibitors, usually engage in a weak-binding interaction with the enzyme [23]. Non-ATP competitive inhibitors do not compete with the high intracellular ATP-concentration and thus offer a distinct pharmacological advantage. Moreover, the involvement of GSK-3 in several essential signalling pathways imposes a limit on the GSK-3 inhibition, complete inhibition will result in adverse events.

Thus GSK-3 inhibitors suitable for AD therapy have to strike a balance between the different pathways. This delicate balance may be achieved by moderate inhibition in combination with excellent pharmacokinetics. Thiadiazolindiones (TDZDs) are non-ATP competitive GSK-3 inhibitors, which delivered a candidate for phase IIb trials recently [24]. The extended phase II trial (60-day treatment) did not reveal adverse effects [25]. However, the majority of the known GSK-3 inhibitors are ATP competitive and target the ATP binding pocket of GSK-3. Several small-molecule inhibitor/GSK-3 complexes can be extracted from the Protein Data Bank (PDB) (PDB codes: 3PUP (15), 1Q4L (25), 1Q3D (25), 1Q41 (25), 1Q3W (25), 1R0E (34), 2OW3 (40), 2JLD (55), 3M1S (56), 1UV5 (65), 3I4B (113), 3F7Z (119), 3F88 (119), 3GB2 (120), 1Q5K (124), 2O5K (127), 3L1S (130), 3Q3B (136), 1I09 (138)). A closer view at the interactions of these inhibitors with GSK-3 will be provided in the following sections. 

## 2. Small-Molecule Inhibitors of Glycogen Synthase Kinase 3

Several ATP competitive GSK-3 inhibitors from different structural classes are highlighted in this paper. The *in vitro* and *in vivo* data are summarized if available. It should be noted that the IC_50_ values strongly depend on assay conditions and thus may vary 100 fold depending on ATP and enzyme concentration as well as incubation time. The interactions between the inhibitors and the ATP binding pocket are depicted.

### 2.1. Lithium Chloride

**Lithium chloride** (**LiCl**) was the first GSK-3 inhibitor to be discovered. However, there are several other biological targets for lithium resulting in adverse events and a rather small therapeutic window. This effectively rules out the use of **LiCl** in the therapy of AD. The mechanism by which **lithium** inhibits GSK-3 is unknown, but two hypotheses were proposed: (a) **lithium** (**Li^+^**) is a competitive inhibitor of GSK-3 with respect to **magnesium** (**Mg^2+^**), but neither competitive to substrate nor to ATP (b) **lithium** inhibits **potassium** deprivation [25–27]. This paper focuses on small organic molecules that target specifically GSK-3, thus the activity of lithium salts will not be reviewed. Also covalent or irreversible inhibitors, like the TDZD **NP-12**, will be noted, but not further discussed as well as the **FRATtide**, a peptide derived from FRAT1, which binds to GSK-3 and blocks GSK-3 from interacting with axin [28, 29]. 

### 2.2. Maleimide Derivatives

**Figure 1:**
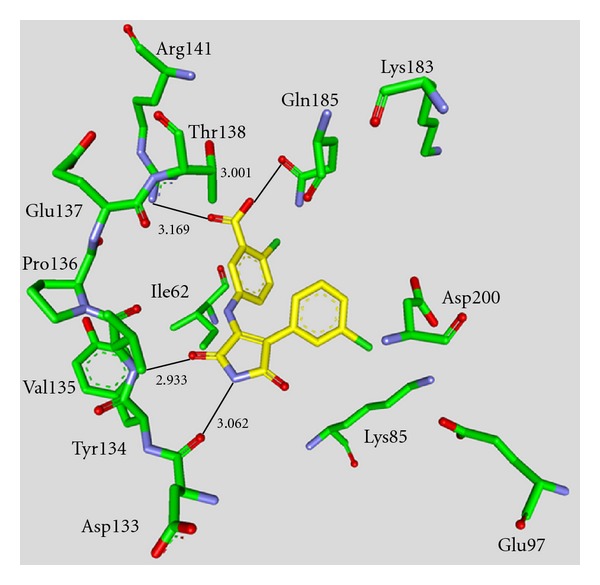
Compound **3** (**I-5**) in the ATP binding pocket of GSK-3*β*; important protein-inhibitor interactions are shown. The distance is denoted by Å. PDB code 1Q4L [31].

**Figure 2:**
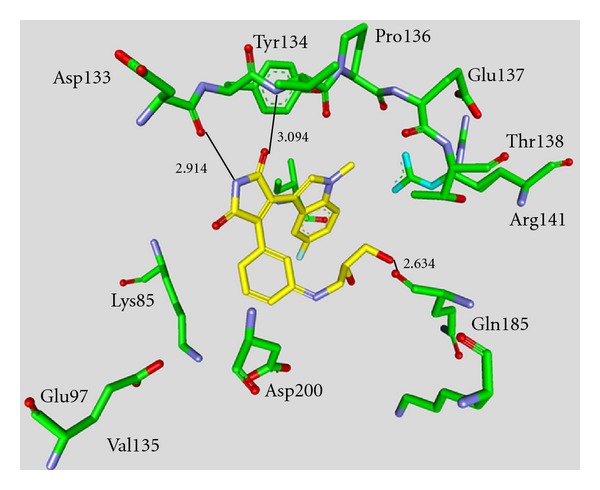
Compound **13** in the ATP binding pocket of GSK-3*β*; important protein-inhibitor interactions are shown. The distance is denoted by Å. PDB code 1R0E [40].

**Figure 3:**
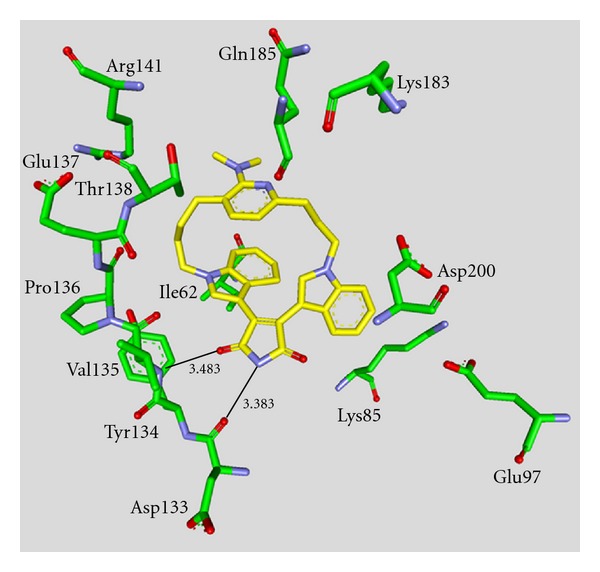
Compound **23** in the ATP binding pocket of GSK-3*β*; important protein-inhibitor interactions are shown. The distance is denoted by Å. PDB code 2OW3 [46].

Maleimide derivatives have been reported as scaffolds for ATP competitive GSK-3 inhibitors. Researchers at SmithKline Beecham Pharmaceuticals reported that 3-anilino-4-arylmaleimides **1**–**3** (Table 1) are potent and selective inhibitors of GSK-3 [30]. The compounds displayed GSK-3 inhibition (IC_50_) at low nanomolar concentrations. The selectivity of compound **2** was evaluated using a panel of 25 kinases. The majority of the kinases showed less than 30% inhibition at an inhibitor concentration of 10 *μ*M. The complex of **3** (named **I-5**) with GSK-3*β* (Figure 1) elucidated the binding mode to the ATP pocket [31]. Herein, the maleimide nitrogen interacts with the carbonyl oxygen of Asp133 and the oxygen of compound **3** with the backbone nitrogen of Val135. Two additional interactions are observed between a carboxylate oxygen and Arg141 and with Gln185. Compound **4** (**SB-216763**) inhibited GSK-3*α* with an IC_50_ of 34 nM (Table 2). Derivative **4** exhibited little or no inhibition of the 24 kinases tested in the panel [32]. Incubation of cerebellar granule neurones with this compound reduced the death rate in a concentration-dependent manner in response to either stimulus. The maximal neuroprotection was observed with 3 *μ*M of **SB-216763 **[33]. A 60% reduction in GSK-3*β* activity levels was observed in the hippocampus, but not in the cortex of **SB-216763** treated animals versus vehicle-treated rats [34]. The compounds **5**–**11** revealed good potencies against GSK-3. Concerning compounds **7** and **9**–**11**, much higher potencies (IC_50_ < 4 nM) were ascertained in comparison to compound **1** (Table 2) [36–38]. The compounds **10** and **11** demonstrated very good GSK-3 inhibition and improved selectivity against PKC*β*II, CDK2, and CDK4 and inhibit tau phosphorylation in a neuronal cell line [38]. Whereas compound **12** was analyzed by *in silico* docking, the binding mode of **13** (IC_50_ = 0.6 nM) was determined by X-ray crystallographic analysis (Figure 2) [40]. Similar to compound **3**, the maleimide nitrogen of **13** interacts with the carbonyl oxygen of Asp133 and its oxygen with the backbone nitrogen of Val135. Furthermore, compound **13** forms a crucial hydrogen bond to Gln185. Maleimide **13** was screened against 317 kinases to provide data on kinase selectivity in order to predict potential safety issues. It was found that compound **13** inhibits only 36 kinases with >90% inhibition at 10 *μ*M [40]. Compounds **14** and **15** are reversible GSK-3 inhibitors in the low micromolar or high nanomolar range and can be used as chemical precursors for the corresponding halomethylketones (HMKs). These HMKs are irreversible inhibitors, they alkylate Cys199, which is located in the ATP binding site of GSK-3 [41]. Further maleimide derivatives are listed in Table 3. All of them, compounds **16**–**27** showed IC_50_/*K*_*i*_ values in the low nanomolar range [42–48]. The majority of these structures was synthesized by Johnson & Johnson Pharmaceutical R & D. The compounds **17** and **18** were evaluated against a broad panel of 55 protein kinases. 10 *μ*M of derivative **17** or **18** inhibited GSK-3*β* kinase activity by 100% in the presence of 10 *μ*M ATP. Compound **18** exhibited excellent selectivity for GSK-3*β* except for the moderate selectivity against PKC*β*II. Meanwhile, the bis-7-azaindolylmaleimide **17** exhibited high selectivity at GSK-3*β* against all kinases tested [43]. The activity of compound **23** was tested in a kinase panel containing 100 diverse protein kinases. An IC_50_ of 3 nM and a 460 fold selectivity for GSK-3*β* over PKC*β*II was reported [46]. The X-ray structure analysis of cocrystallized compound **23** and GSK-3*β* is illustrated in Figure 3.

The maleimide establishes key hydrogen bond contacts with the residues Asp133 and Val135. The compounds **25** and **26** were docked into the ATP binding site of GSK-3*β* (PDB code 1R0E). Similar to other maleimide derivatives, the maleimide nitrogen interacts with the carbonyl oxygen of Asp133 and the oxygen with the backbone NH of Val135. In addition to these main interactions, the hydroxymethyl group of compound **26** interacts with the side chain of Arg141 [48].

The benzo[e]isoindole-1,3-dione **28** (Table 4) displays an IC_50_ of 304 nM in the presence of 100 *μ*M ATP. It was evaluated in a panel of 22 representative kinases. 5 *μ*M of compound **28** inhibited 91% of GSK-3*β* activity as well as 71% of CDK2/cyclin A and 53% of KDR (VEGFR2) [49]. The inhibition of GSK-3 leads to ectopic activation of the Wnt pathway during zebrafish development, resulting in a headless embryo. Thus, zebrafish embryos, which were treated with compound **28**, had highly restricted brain defects that ranged from smaller eyes and forebrain to a complete loss of these structures [49]. Compound **29** displayed a lower IC_50_ *in vitro* than **30**, whereas compound **30** displayed better *in vivo* efficacy. 25 *μ*M of derivative **30** resulted in the eyeless phenotype of zebrafish embryos after 3 days of incubation [50]. The synthetic activity dedicated to this structural class has been strong and is ongoing [51–55]. 

### 2.3. Staurosporine and Organometallic Inhibitors

**Figure 4:**
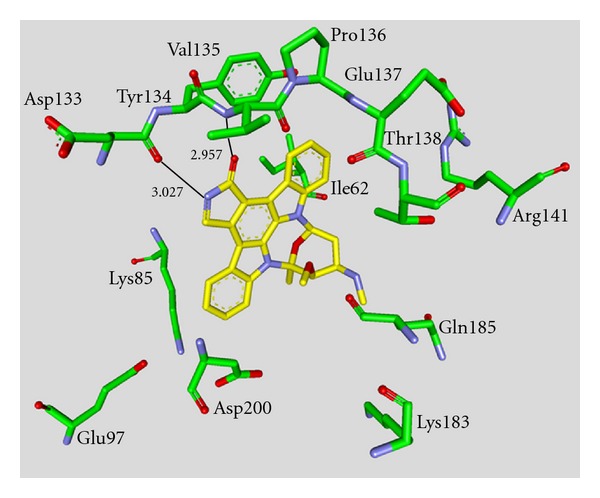
Compound **31** in the ATP binding pocket of GSK-3*β*; important protein-inhibitor interactions are shown. The distance is denoted by Å. PDB code 1Q3D [31].

**Figure 5:**
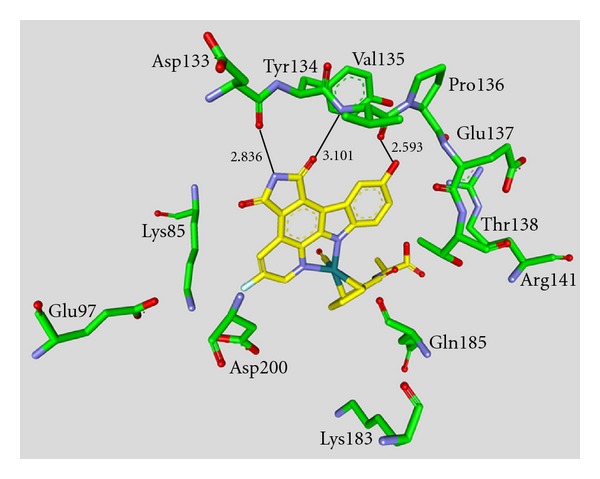
Compound **34** in the ATP binding pocket of GSK-3*β*; important protein-inhibitor interactions are shown. The distance is denoted by Å. PDB code 2JLD [61].

**Figure 6:**
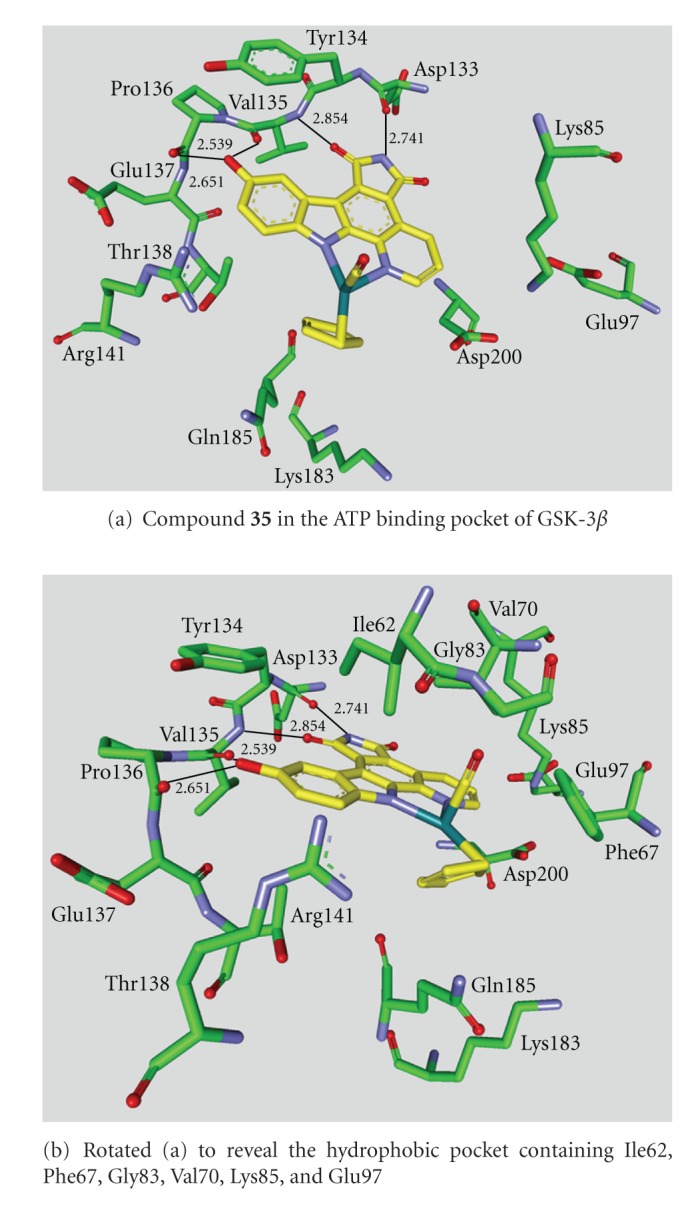
Compound **35 **in the ATP binding pocket of GSK-3*β*; important protein-inhibitor interactions are shown. The distance is denoted by Å. PDB code 3M1S [62].

**Figure 7:**
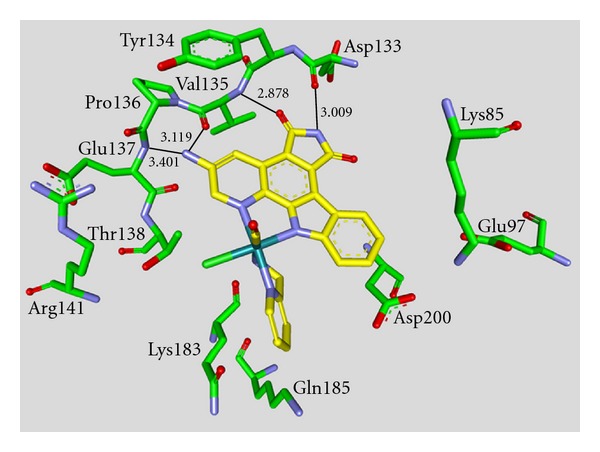
Compound **36 **in the ATP binding pocket of GSK-3*β*; important protein-inhibitor interactions are shown. The distance is denoted by Å. PDB code 3PUP [22].

**Staurosporine** (**31**) is a natural product from the bacterium *Streptomyces staurosporeus *[56]. It exerts antimicrobial, hypotensive, and cytotoxic activity [57]. **Staurosporine** (**31**) is also a potent GSK-3*β* inhibitor with a reported IC_50_ value of 15 nM (Table 5) [58]. The cocrystal structure of **Staurosporine** (**31**) with GSK-3*β* is elucidated in Figure 4. Again, the maleimide **31** interacts with the Asp133 carbonyl oxygen and the backbone nitrogen of Val135. These are the only direct hydrogen bonds observed between GSK-3*β* and this inhibitor [31]. The other polar interactions, for example, water-mediated interactions are not denoted. The organometallic ruthenium complex **32** is a remarkably potent inhibitor of GSK-3 (Table 5). The IC_50_ for GSK-3*α* is 3 nM and 10 nM for GSK-3*β*. The compound was evaluated against 10 kinases and displayed activity against GSK-3 and RSK1 (IC_50_ = 100 nM) only. The authors observed a water-mediated contact between the carbonyl ligand and the carbamide of Gln185 and assumed that this contributes to the specificity of GSK-3 [59]. Compound **33** showed improved selectivity. A test panel of more than 50 kinases was not significantly inhibited at 100 nM. Compound **33** was reported to activate the Wnt signaling pathway in cell culture experiments at nanomolar concentrations. The treatment of zebrafish embryos in early development by compound **33** caused malformations, for example, a head structure lacking the eyes and a stunted tail [60]. The S-diastereomer of compound **34 **is a weaker GSK-3*β* inhibitor than the R-diastereomer: IC_50_ of 0.22 nM at 100 *μ*M. Compound **34** (R-diastereomer, Table 5) exerts the highest reported potency against GSK-3*β* with an IC_50_ of 40 pM. Unfortunately, the evaluation in a kinase panel was not reported yet [61]. The complex of derivative **34** with GSK-3*β* (Figure 5) provided insight into the binding mode in the ATP pocket of GSK-3*β*. The interactions between the imide NH and the carbonyl oxygen of Asp133 and between the imide carbonyl and the NH of Val135 are typical. Another interaction is marked between the carbonyl oxygen of Val135 and the indolyl OH. The compound **34**, and the analogues **35** (**(*R*)-DW 12**) and **36** (**Λ-OS1**) contain a carbonyl, which interacts with the flexible glycine-rich loop of GSK-3*β* (Figures 6 and 7). Thereby Ile62, Gly63, Phe67 and Val70 form a small hydrophobic pocket (Figure 6(b), compound **35**). The structure activity analysis of ruthenium-based GSK-3 inhibitors lacking the carbonyl revealed the contribution of this carbonyl to potency and selectivity [22, 61, 62]. 

### 2.4. Indole Derivatives

**Figure 8:**
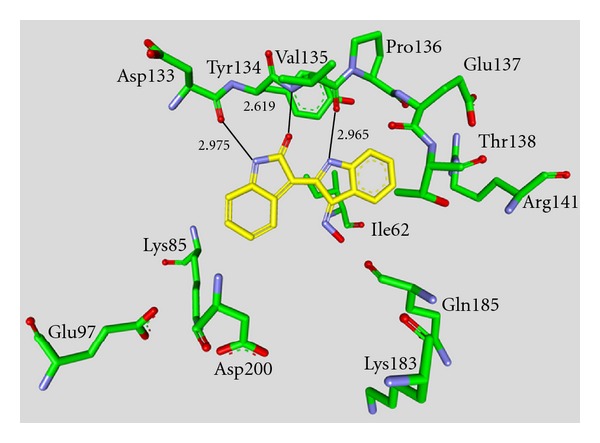
Compound **37** in the ATP binding pocket of GSK-3*β*; important protein-inhibitor interactions are shown. The distance is denoted by Å. PDB code 1Q41 [31].

**Figure 9:**
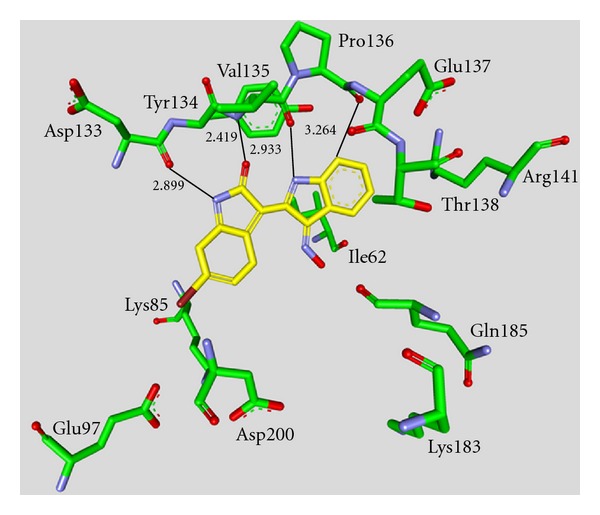
Compound **39 **in the ATP binding pocket of GSK-3*β*; important protein-inhibitor interactions are shown. The distance is denoted by Å. PDB code 1UV5 [63].

Several pharmaceutical companies have reported and patented indole derivatives as GSK-3 inhibitors [67–74]. Indirubins are likewise indole derivatives. They are related to the naturally occurring indigo dyes, and their pharmacological properties have long been known in traditional Chinese medicine, for example, in the treatment of leukemias [15]. Except for the dye indirubin **38**, all other noted indole derivatives (**37**, **39**–**50**) revealed an IC_50_ below 100 nM. Compound **37**, the **indirubin-3′-monoxime**, was reported to display an IC_50_ of 22 nM on GSK-3 inhibition (Table 6). This unselective compound is a strong inhibitor of the closely related kinases CDK1 and CDK5 [58]. Compound **37** did not affect p-tau levels in neither cortex nor hippocampus of P12 rats despite the high concentration of the compound in the brain (13 *μ*M) [34]. Three important interactions of compound **37** and GSK-3*β* are displayed in the X-ray in Figure 8 [31]. Maternal Wnt activity is necessary for dorsal axis formation in *Xenopus laevis* embryos, whereas head formation requires the inhibition of zygotic Wnt activity. The *Xenopus laevis* embryos were treated with compound **39 **(**BIO**) or **LiCl**, respectively; in order to challenge these two GSK-3 regulated events *in vivo*. **LiCl** treatment leads to a hyper dorsoanteriorization at the expense of trunk and tail when applied during early cleavage stage. **39** exerted the same effect on the embryos. Compound **39** inhibited GSK-3 with an IC_50_ of 5 nM, but also CDK1, CDK2, and CDK5 in a nanomolar range [63, 64]. The binding mode of **39** in the ATP pocket of GSK-3*β* has been determined by X-ray crystallographic analysis. The four major interactions between the inhibitor and GSK-3*β* are shown in Figure 9. The nitrogen interacts with the carbonyl oxygen of Asp133 and the oxygen with the backbone nitrogen of Val135. Moreover, interactions with the oxygens of Val135 and Pro136 as well as the van der Waals contact between the bromine and Leu132 were observed. Compounds **41 **and** 43 **are potent GSK-3 inhibitors and very selective against CDK1/cyclin B and CDK5/p25 [64]. In 2007, this class of indirubine derivatives was patented by Meijer et al. [75]. This patent lists *in vivo* activity for several compounds. The derivatives **44**–**46** feature an extended amino side chain at position R^1^. They were prepared to enhance selectivity and water solubility versus compound **39**. These compounds were reported to inhibit *β*-catenin Ser33/37/Thr41 phosphorylation by GSK-3. Compounds **44**–**46** were also less cytotoxic than compound **39 **in the MTS reduction assay of SH-SY5Y neuroblastoma cells [65]. The compounds **47**–**50** are potent, yet unselective GSK-3 inhibitors [66].

### 2.5. Paullone Derivatives

Paullones have been reported as potent ATP competitive inhibitors of CDKs and GSK-3*β* [76, 77]. Compounds **51**–**55** revealed that a defined derivatisation of one substituent only can increase the GSK-3*β* inhibition up to 155 fold. Alsterpaullone **55** (**9-nitro-paullone**) is one of the most potent GSK-3*β* inhibitors and competes with ATP for binding to GSK-3*β* (Table 7). 

**Figure 10:**
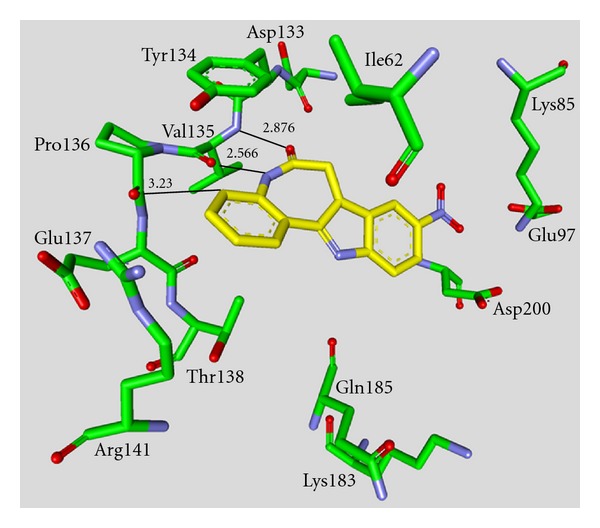
Compound **55** in the ATP binding pocket of GSK-3*β*; important protein-inhibitor interactions are shown. The distance is denoted by Å. PDB code 1Q3W [31].

Compound **55 **was evaluated in a kinase panel with 25 kinases and exhibited high selectivity for GSK-3*α*/*β*, CDK1/cyclin B, CDK2/cyclin A, CDK2/cyclin E, and CDK5/p35. All measured IC_50_ values were in the nanomolar range. Alsterpaullone **55** was reported to inhibit the *in vivo* phosphorylation of tau at AD-specific sites by GSK-3*β* [76]. Paullone **55** formulated in 20% DMSO/25% Tween-80 and injected *s.c.* led to a reduction in 43 kDa tau phosphorylation in cortex after 2 h, though not in the 49 kDa isoform. Furthermore, the phosphorylation levels of the 43 kDa and 49 kDa isoforms in the hippocampus were significantly decreased [34]. Most of the paullone derivatives were patented by Meijer and Kunick in 2001 [81]. The binding mode of **55 **has been determined by X-ray crystallographic analysis (Figure 10) [31]. The interactions of alsterpaullone and GSK-3*β* include two hydrogen bonds with Val135 and one interaction between the nitro group and the side chain amino group of Lys85. The compounds **56 **and** 57** inhibited GSK-3 as well as CDK1/cyclin B and CDK5/p25 in the nanomolar range [78]. The exchange of nitrogen and carbon in compound **58 **and **59** decreased the inhibitory activity, but provided selectivity to **58, **which is void of CDK-inhibitory effects [79]. However, compound **58 **was tested against three kinases (GSK-3*β*, CDK1/cyclin B, and CDK5/p2) only. The derviatisation of the R^2^ motif, see compounds **57** and **60**–**64** in Table 7, leads to the most potent inhibitor **64**. Compound **64**, with an IC_50_ of 0.8 nM is likewise nonselective for GSK-3. It preferentially inhibited the CDK/GSK-3 family in the nanomolar range and VEGFR-2, VEGFR-3, and Src in the submicromolar range [80].

### 2.6. Pyrazolamide Derivatives

GlaxoSmithKline identified another class of GSK-3 inhibitors in 2003 (Table 8). The precursor was identified by a pharmacophore search of the in house database. The compounds **65**–**67** were profiled against a panel of 25 kinases including GSK-3*β*. An excellent selectivity was obtained against the majority of the kinases. However, a significant inhibition of CDK2/cyclin A was reported [82]. Compounds** 68** and **69** displayed improved potency, but nevertheless inhibited CDK2/cyclin A. Only compound **70** showed an excellent GSK-3 potency and improved CDK-2/cyclin A selectivity [83]. The IC_50_ comparison of compounds **71**–**73** revealed that the Ph-4-OH motif is the best. The most potent inhibitor **74 **(IC_50_ = 0.8 nM) was tested against a panel of kinases and showed a reduction in the overall selectivity profile [84, 85]. Compounds **75**–**77 **showed an excellent selectivity against CDK-2/cyclin A. In addition, compound **77 **demonstrated an excellent overall selectivity profile against all kinases of the panel [85]. Unfortunately, no further studies were published concerning this series. 

Takeda Pharmaceutical disclosed compound **78** in 2009. It is a very potent inhibitor of GSK-3 with an IC_50_ of 2.3 nM for GSK-3*α* and 2.0 nM for GSK-3*β*. It had no inhibitory effect on 23 kinases, and only a weak inhibition was detected for CDK1/cyclin B, CDK2/cyclin A, CDK5, and JNK1. The cold-water stress model (CWS) with male C57BL/6Njcl mice was used to evaluate the *in vivo* efficacy of compound **78**. CWS transiently induces *in vivo *tau hyperphosphorylation as reported in previous studies. The compound displayed highly potent inhibition of *in vivo* phosphorylation in CWS mice and reduced sarkosyl insoluble tau in old homozygous JNPL3 mice. Furthermore, compound **78** inhibited tau phosphorylation at GSK-3-directed sites in rat primary neuronal cells and mouse brain tissue [86]. Several structures containing a pyrazole core have shown promising results in animal models of diabetes, for structural information see denoted references. 

The pyrazolamides developed by Wyeth-Ayerst Research, now Pfizer, showed significant plasma glucose-lowering activity (16–42% reduction) in genetically obese, diabetic db/db mice [87, 88]. Novartis published a series of compounds with ED_50_ values for glucose reduction in ob/ob mice of 3.0 mg/kg/day [89]. In 2011, the Merck Research Laboratories disclosed a potent human glucagon receptor antagonist with good pharmacokinetic profiles in four preclinical species. One of these compounds showed excellent oral pharmacodynamic efficacy in rhesus monkeys and transgenic mice by blocking glucagon-induced hyperglycemia [90]. 

### 2.7. Pyrimidine and Furopyrimidine Derivatives

**Figure 11:**
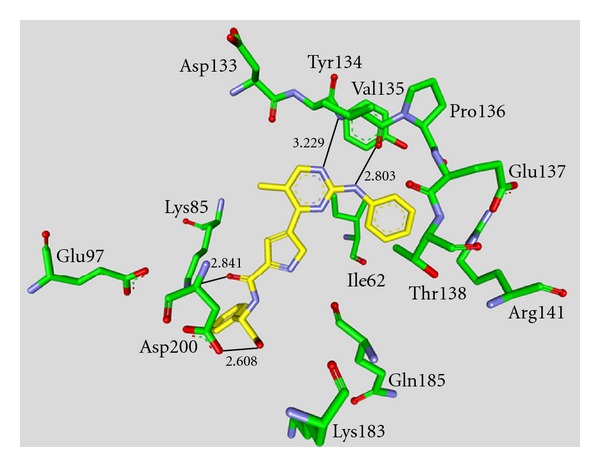
Compound **94** in the ATP binding pocket of GSK-3*β*; important protein-inhibitor interactions are shown. The distance is denoted by Å. PDB code 3I4B [93].

GSK-3 inhibitors bearing the pyrimidine moiety are listed in Tables 9 and 10. This series is characterized by a high number of patent applications, particularly by Vertex Pharmaceuticals [97–122]. Compounds **79** and **81 **inhibited GSK-3*β* in the nanomolar range. The *in silico* docking of compound **80** into the ATP binding pocket of GSK-3*β* suggested that **80** makes two hydrogen bonds with the hinge region and one interaction with the positively charged Arg141 [28, 91]. Unfortunately, the activity of these compounds in a kinase panel was not disclosed. The compounds **82**–**90** are weak inhibitors of GSK-3, and there are no public data for *in vivo* activity nor selectivity. Docking of **90** into the PDB structure 1Q5K of GSK-3*β* suggested that one nitrogen and the secondary amine form hydrogen bonds to the hinge region at the Val135 NH and carbonyl, respectively. The following compounds **91**–**93** showed IC_50_ values below 100 nM, but **91** and **92 **inhibit Aurora A in the nanomolar range. Derivative **93** (GSK-3*α* IC_50_ = 61 nM, GSK-3*β* IC_50_ = 41 nM) inhibited Aurora A at micromolar concentration only. *In vivo* studies of this compound in rats showed 34% oral bioavailability and good exposure [92]. Compound **94**, a lead compound for ERK2 inhibition, is a nonselective GSK-3 inhibitor and inhibited all 5 kinases in the panel, which included GSK-3 [93]. The complex of compound **94** with GSK-3*β* (Figure 11) provided the binding mode in the ATP pocket of GSK-3. The secondary amine interacts with the carbonyl oxygen of Val135 and the nitrogen of compound **94** with the backbone NH of Val135. Two additional interactions are present between OH and Asp200 and between the carbonyl and the primary amine of Lys85. The arylimidazoles **95 **(**CHIR 99021**) and **96 **(**CHIR 98014**) are very effective ATP competitive inhibitors of murine and rat GSK-3 (IC_50_ ≤ 10 nM). Both compounds exhibited 500 to 10000 fold selectivity for GSK-3 versus 20 other kinases tested. These GSK-3 inhibitors rapidly lower blood glucose levels in diabetic rodent models and enhance glucose transport as well as GS activation in insulin-resistant oxidative skeletal muscle from type 2 diabetic rats [94]. The nitropyridine **96** exerts a very potent reduction of Ser396 tau phosphorylation in a human neuronal cell line. P12 rats were injected *i.v.* with 30 mg/kg of **CHIR 98014** (**96**, dissolved in DMSO) to test the efficacy on tau phosphorylation *in vivo*, resulting in a maximal brain concentration of 7 *μ*M. Tissue analysis by Western blotting using a p-tau Ser396 antibody showed approximately 40% reduction in the phosphorylation of 43 kDa and 49 kDa tau in the cortex and a significant 3 fold reduction of the 43 kDa isoform in the hippocampus. Moreover, a 50% reduction of GSK-3*β* activity was observed in compound **96 **treated animals versus vehicle [34].

The denoted furopyrimidines **97**–**102** inhibited GSK-3*β* with an IC_50_ below 40 nM (Table 10). Compound** 100** (GSK-3*β* IC_50_ = 5 nM) displayed excellent selectivity against 25 kinases, including CDK2/cyclin A, which was inhibited with an IC_50_ of 0.46 *μ*M. Compound **100** was examined in a glycogen accumulation assay in L6 cells and exhibited excellent induction of glycogen accumulation (EC_50_ = 0.39 *μ*M) [95]. Compound **101** was also profiled by cross-screening against a variety of kinases and showed an excellent overall selectivity against all kinases tested including CDK2. The docking of **101** into the ATP binding site of GSK-3*β* provided a likely interaction. One nitrogen and NH_2_ of aminopyrimidine are anchored to the carbonyl moiety and NH of Val135 via hydrogen bond interactions. The 3-pyridine moiety is located close to Lys85 of the conserved salt bridge (Lys85/Glu97). The EC_50_ value of compound **101** in the glycogen accumulation assay in L6 cells was 3.2 *μ*M and thus 9 fold higher than for compound **100 **[96].

### 2.8. Oxadiazole Derivatives

**Figure 12:**
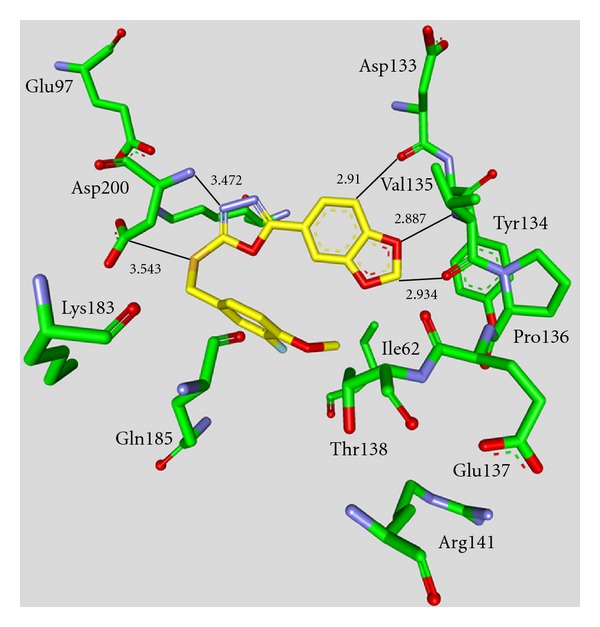
Compound **110** in the ATP binding pocket of GSK-3*β*; important protein-inhibitor interactions are shown. The distance is denoted by Å. PDB code 3F7Z [125].

**Figure 13:**
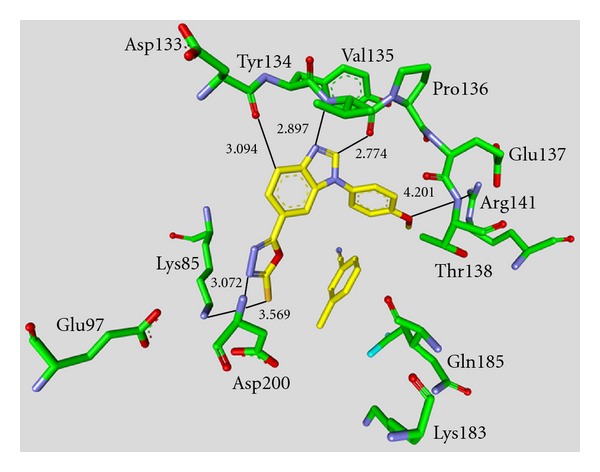
Compound **114** in the ATP binding pocket of GSK-3*β*; important protein-inhibitor interactions are shown. The distance is denoted by Å. PDB code 3F88 [125].

**Figure 14:**
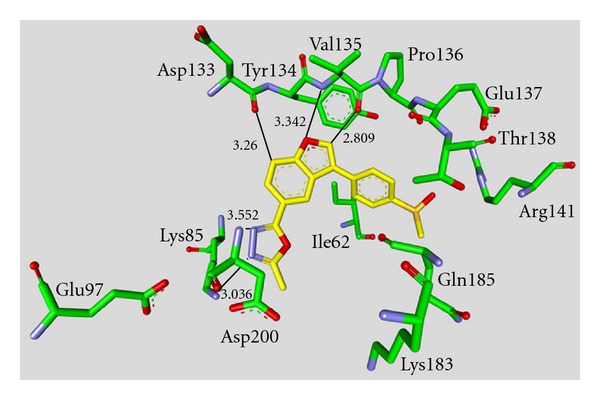
Compound **116** in the ATP binding pocket of GSK-3*β*; important protein-inhibitor interactions are shown. The distance is denoted by Å. PDB code 3GB2 [126].

Tables 11–13 list GSK-3 inhibitors featuring the oxadiazole moiety. The depicted 1,2,5-oxadiazoles, **103**–**109**, revealed IC_50_ values from 0.1 *μ*M to more than 1.1 *μ*M. Compounds **104**–**106** were screened for inhibitory activity against a panel of 32 kinases at 100 *μ*M ATP concentration. All compounds gave at least 100 fold selectivity for GSK-3 compared to CDK2. However, 10 *μ*M of compound **104** inhibited other kinases like MSK1 and DYRK1A, the latter activity is of interest for neurodegenerative diseases. The above mentioned compounds **104**–**106** displayed both sufficient cell penetration and suitable water solubility [123]. The dioxolane **110** was identified as GSK-3*β* inhibitor (IC_50_ = 65 nM; Table 12) by high throughput screening. The X-ray analysis of the GSK-3*β* cocrystallized compound confirmed the interaction with the ATP binding site (Figure 12). One oxygen and a neighbouring hydrogen atom of the benzodioxole establish hydrogen bonds with the amide NH hydrogen and carbonyl oxygen of Val135 in the hinge region. The nitrogen atoms of the oxadiazole engage in an unique hydrogen bond relay network between Lys85-Glu97-Asp200 via two water molecules (interactions not shown). Further interactions are denoted in Figure 12. After an extensive derivatisation, for example, **111**–**114**, compound **114 **(Figure 13) exerts a 28 fold increased activity (GSK-3*β* IC_50_ = 2.3 nM) compared to its homologue **110** (Table 12). The selectivity of **114** was evaluated in a panel of more than 20 kinases to reveal more than 1000 fold selectivity against CDK1, CDK2, and CDK5. In addition, rat cassette dosing experiments of compound **114 **were performed, which revealed low oral bioavailability. The cocrystal structure of **114** bound to GSK-3*β* was not fully characterized due to the cleavage of the S–C bond in the X-ray beam (Figure 13) [125]. The cocrystal structure indicated that the nitrogen of the benzimidazole forms a hydrogen bond with the backbone NH of the hinge region at Val135, and one nitrogen of the oxadiazole ring makes a hydrogen bond with the NH of Asp200. The hydrogen atom on the carbon of the benzimidazole made an additional hydrogen bond with the carbonyl oxygen of Val135 [125]. The compounds **115** and **116** are enantiomers just as compounds **117** and **118**. The S-isomers **116** and **118** were found to be eutomers. It was reported that the S-isomers possessed good oral absorption in nonfasted Crl: CD(SD)IGS rats with a bioavailability of 72.8% for derivative **116** and 65.5% for derivative **118**. Furthermore, the compounds exhibited favourable blood-brain barrier (BBB) permeability. Compounds **116** and **118** were tested for inhibitory activity against more than 20 kinases and displayed no significant activity. This indicates that these compounds are highly selective GSK-3 inhibitors. The binding mode of **116** in the ATP pocket of GSK-3*β* has been determined by X-ray crystallographic analysis (Figure 14) [126]. One of the nitrogen atoms of the oxadiazole was revealed to interact via a hydrogen bond with the side chain of Lys85 and the other with Asp200. The oxygen atom and one carbon hydrogen of the benzofuran ring interact with the main chain of Val135. Another interaction was observed between one carbon hydrogen and the carbonyl of Asp133. Derivatives **116** and **118** were tested *in vivo* using the CWS model in mice. Here, tau phosphorylation was induced at several GSK-3*β*-directed sites such as Ser199, Thr205, Thr231, and Ser396 in mice. As reported, tau phosphorylation was significantly reduced by 35% for compound **116** and 38% for compound **118 **[126]. Compound **116** was further investigated and revealed significantly decreased hippocampal tau phosphorylation as well as suppression of tau pathology without affecting amyloid *β* pathology [127].

A multistage virtual screening of 289903 molecules resulted in 59 hits of potential GSK-3*β* inhibitors, for example, compound **119**. Biological tests confirmed the IC_50_ of 17 nM and good selectivity versus CDK2. It crosses the BBB and has a good hepatic glycogen effect in mice. The derivatisation, for example, **120–122**, of compound **119** lead to compound **122** with an IC_50_ of 7 nM. Surprisingly, docking experiments of compound **122** revealed a different binding mode in comparison to the cocrystallized oxadiazoles **110**, **114** and **116**. The *in silico* docked oxadiazole motif coordinates to the GSK-3*β* hinge region, Val135, Tyr134, and Asp133, instead of binding to the polar region, Lys85, Cys199, and Asp200 [128].

The last denoted oxadiazoles are the 1,2,4-oxadiazoles **123**–**127**, which exert moderate inhibition of GSK-3*β* only (Table 13). A subset of these oxadiazoles (**123–125**) exhibited weak inhibition of Pim-1 and no detectable inhibition towards seven other kinases tested at 10 *μ*M concentration [129]. 

### 2.9. Thiazole Derivatives

**Figure 15:**
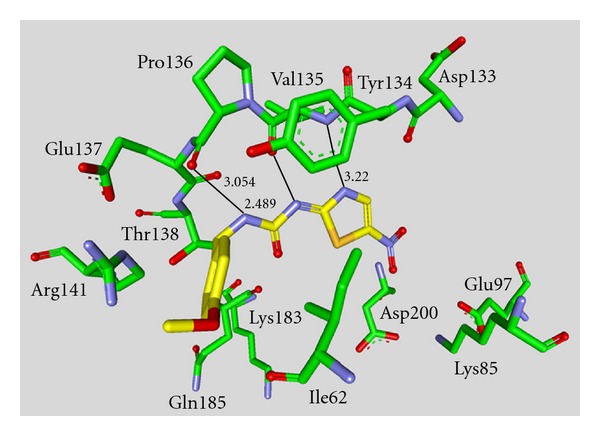
Compound **130** in the ATP binding pocket of GSK-3*β*; important protein-inhibitor interactions are shown. The distance is denoted in Å. PDB code 1Q5K [131].

The benzothiazoles **128** and **129 **(Table 14) are nonselective GSK-3 inhibitors and showed moderate activity against CDKs [130]. This stands in contrast to the thiazolylurea **130** (**AR-A014418**), which strongly inhibited GSK-3 (IC_50_ = 104 nM) but not any other kinase in the panel. **AR-A014418** inhibited tau phosphorylation in transfected 3T3-fibroblasts in a dose-dependent fashion exhibiting an IC_50_ of 2.7 *μ*M. The neuronal loss was reduced in the organotypic culture (N2A cells) and compound **130** by itself did not affect neuronal viability. The cocrystal structure analysis of **AR-A014418** and GSK-3*β* revealed that this compound binds to the hinge region via three hydrogen bond interactions (Figure 15) [131]. Furthermore, compound **130** significantly reduced insoluble tau levels in the brainstem of JNPL3 mice when compared with vehicle treated animals [132]. Surprisingly, another research group reported that **AR-A014418** showed no effect on the phosphorylation levels of neither 43 kDa nor 49 kDa tau in the cortex or hippocampus of postnatal model rats [34]. Novel compounds based on the scaffold of **AR-A014418** were synthesized recently. They showed improved *in vitro* activity and reduced toxicity in the wildtype zebrafish embryo assay [133].

### 2.10. Miscellaneous Heterocyclic Derivatives with GSK-3 Activity

**Figure 16:**
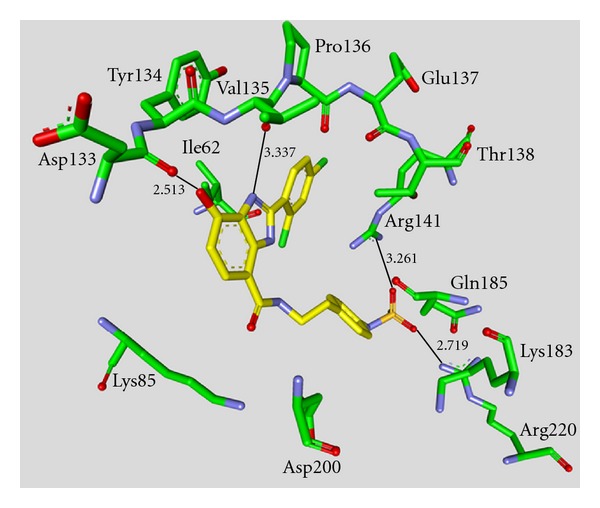
Compound **134** in the ATP binding pocket of GSK-3*β*; important protein-inhibitor interactions are shown. The distance is denoted in Å. PDB code 2O5K [134].

Table 15 lists GSK-3 inhibitors featuring a benzimidazole core. There are neither *in vivo *assays nor selectivity data published for these potential metal chelators **131–134** (Table 15(a)). However, compound **134** (GSK-3*β* IC_50_ = 15 nM) was cocrystallized with GSK-3*β* (Figure 16) [134]. 

Herein, the secondary amine interacts with the carbonyl oxygen of Val135 and the phenolic OH of **134** with the carbonyl of Asp133. Two more interactions are established between the SO_2_ of compound **134 **and the arginines Arg141 and Arg220. The 1-aza-9-oxafluorenes **135**–**137** are moderately active GSK-3*β* inhibitors, but showed activity for CDKs (Table 15(a)) [135].

There is no report for an *in vitro* IC_50_ of the synthetic xanthine **propentofylline** (PPF, compound **138**, Table 15(a)), but studies in the Tg mouse model of AD indicated that PPF exerts a dual effect: reduction of both pathological amyloidogenesis and tau phosphorylation while reducing the ratio of activated versus inactivated GSK-3*β* [136]. 

**Figure 17:**
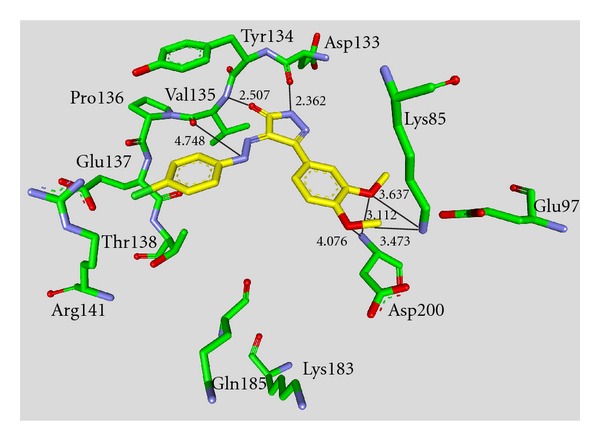
Compound **142 **in the ATP binding pocket of GSK-3*β*; important protein-inhibitor interactions are shown. The distance is denoted in Å. PDB code 3L1S [137].

The pyrazolone **139** was identified as potential scaffold in a compound screen for novel GSK-3 inhibitors. Derivatisation has increased the potency of the derivatives (*K*_*i*_ from 1.49 mM to 0.8 nM (Table 15(b)). The compounds **140**, **141**, and **143** showed selectivity in a kinase panel of 14 kinases. A cocrystallization was realized with compound **142** (Figure 17) and provided the interactions of the pyrazole moiety with the GSK-3 backbone aminoacids Asp133 and Val135. In addition, the methoxy substituents make hydrogen-bonding contacts with both Asp200 and Lys85. These interactions and the hydrophobic contacts of the phenyl rings are thought to be responsible for the potency and selectivity [137]. A recent docking study of the pyrazolone **144** confirmed the interactions of compound **142 **with GSK-3*β*. It is an active (IC_50_ of 34 nM) and selective (kinase panel of 40 kinases) structural analogue to the previously described maleimides **9**–**27** (Tables 2 and 3). Furthermore, compound **144** was evaluated in a model of oxidative stress induced by homocysteic acid and displayed full neuroprotective activity at 1 *μ*M. Additionally, it was able to reduce locomotor activity in the chlordiazepoxide/amphetamine-induced hyperactivity model *in vivo *[138]. The compounds **145**–**147** are moderate GSK-3 inhibitors and were not tested against other kinases to evaluate their selectivity (Table 15(b)). Compound **145** increased the glycogen content in the liver of Sprague-Dawley rats in a dose-dependent manner [139].

**Figure 18:**
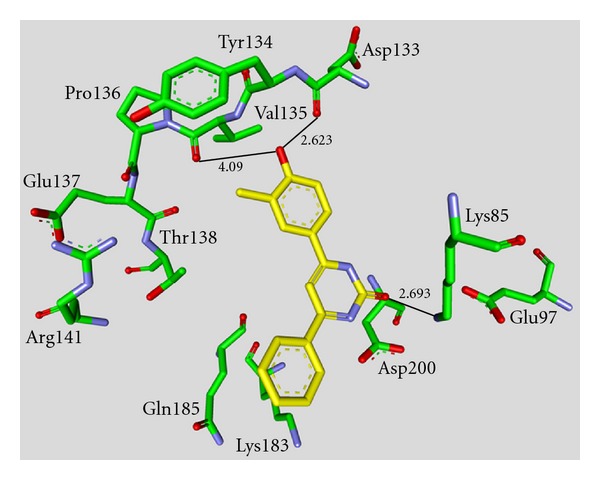
The HTS hit named **Compound 1 **in the ATP binding pocket of GSK-3*β*; important protein-inhibitor interactions are shown. The distance is denoted in Å. PDB code 3Q3B [140].

**Compound 1** is a HTS hit, which was cocrystallized with GSK-3*β* and solved by X-ray crystallography (Figure 18). The overlap of the inhibitor **Compound 1** with a pyridone derivative revealed interactions with the catalytic triad, especially Lys85, and the protein backbone aminoacid Val135 of GSK-3*β* [140]. This leads to the latest ATP competitive GSK-3 inhibitors, the pyridones **148**–**150** (Table 15(b)). Compound **150** revealed potent *in vitro* inhibition with an IC_50_ of 16.1 nM against GSK-3*β*. It was selective in a broad panel of kinases, including CK2 and CLK1. Compared to compound **148**, compound **150** exhibited an improved *in vitro* human liver microsome intrinsic clearance value of 16.3 mL/min/kg. The *in vivo* CNS penetration assay demonstrated a good free brain to free plasma ratio and the assessment of potential genetic toxicology hazards was negative for compound **150 **[140]. Last but not least, there were several GSK-3 inhibitors isolated from marine organisms. These alkaloids have the potential to provide new scaffolds comparable to the established indirubines. Manzamines, meridianins, hymenialdisine and dibromocantharelline inhibit GSK-3*β* in the *μ*M range and display promising selectivity and *in vivo* results (Table 15(c); compounds **151**–**154**) [141–144]. 

## 3. Activity and Selectivity Profiling

**Figure 19:**
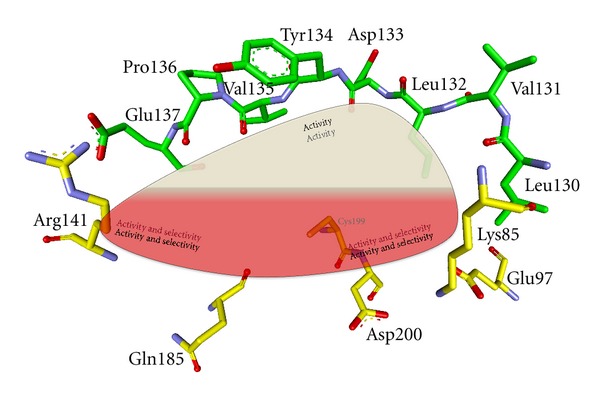
Schematic view in the ATP binding pocket of GSK-3*β*; important areas for activity and selectivity are denoted by PDB code 1I09 [146].

A plethora of GSK-3 inhibitors was discovered in recent years, and most of these displayed good-to-excellent inhibition of this kinase. However, selectivity and safety against other kinases remains to be a challenge. The structural analysis of the ATP competitive inhibitors may guide the development of more selective GSK-3 inhibitors. All ATP competitive inhibitors establish hydrogen bonds with the backbone atoms of Asp133 and Val135. Contact to these aminoacids is a key to enhance affinity to GSK-3, but it does not provide selectivity over other kinases. Moreover, Pro136 appears in several complexes to strengthen the interaction of the inhibitor with the backbone (Figure 19). One region of GSK-3 may offer privileged access to enhanced activity and selectivity: it is the region characterized by the aminoacids Lys85, Glu97, and Asp200. Lys85 was observed to form a salt-bridge with Glu97 and simultaneously with Asp200, which is expected to be less significant and potent as the one with Glu97 [145]. The interaction of an inhibitor with this region holds potential to increase activity and selectivity for GSK-3. This interaction can be mediated via water molecules, as observed for **AR-A014418** (**130**), or be established by direct contact. We observed in our dataset that a direct interaction with this region may cause a loss of selectivity (data not shown). There is a salt-bridge formed by Glu137 and Arg141 in the entrance area of the ATP pocket of GSK-3*β*. This region seems to contribute to the activity and selectivity of several inhibitors. 

Another important interaction between GSK-3 and an organometallic inhibitor was reported by Bregman et al. They observed a water-mediated contact between the carbonyl ligand of the inhibitor and the carboxylate of Gln185 [59]. This water molecule was also found in other GSK-3*β* inhibitor complexes and seems to be responsible for an increased activity and especially for an improved selectivity towards GSK-3. 

Docking studies with our inhibitors confirmed this interaction and further explained the selectivity of these inhibitors (data not shown). Feng et al. observed that the ruthenium-coordinated CO ligand of their inhibitor interacts with the flexible glycine-rich loop formed by Ile62, Gly63, Phe67, and Val70. This pocket, which is not shown in Figure 18, seems to be crucial for potency and selectivity [22]. Figure 18 illustrates in a simple scheme how to enhance activity and selectivity of inhibitors. The interaction with at least two of the three areas deems necessary to provide active and selective inhibitors. 

## 4. Examples of *In Vivo* Tests

Over the last decade, several animal models have been developed to study tauopathies and other neurodegenerative disorders *in vivo*. Despite some obvious advantages of the diverse AD invertebrate systems, the vertebrate animal models of AD are generally favoured (for detailed invertebrate reviews, see [147–149]). Vertebrate models are evolutionary and morphologically closer related to humans, which makes the direct translation of experimental results easier and more reliable. A conditionally GSK-3*β* overexpressing mouse was reported by Lucas et al. in 2001. They demonstrated that GSK-3*β* overexpressed *in vivo* results in neurodegeneration and proposed that these mice can be used to study some aspects of AD [150]. In 2005, Perez et al. and Ribe et al. developed and characterized a double-transgenic mouse line based on overexpression of human mutant APP and tau [151, 152]. They treated this transgenic mouse model with **NP12**, a non-ATP competitive GSK-3*β* inhibitor, and observed lower levels of tau phosphorylation, decreased amyloid deposition and prevention of memory deficits [18]. The APP-V7171 × Tau-P301L mice with combined amyloid and tau pathology and the GSK-3*β*  × Tau-P301L mice with tauopathy only were reported by Terwel et al. in 2008. These models offer the possibility to explore molecular signals that act upstream and downstream of, or in parallel with GSK-3 isozymes [153]. Two transgenic mice models were developed to study the interaction between APP or A*β* and tau: the triple-transgenic model (3 × Tg-AD) harboring PS1_M146V_, APP_Swe_ and tau_P301L_ transgenes and the TAPP mice [154, 155]. 

The transgenic mice have been the major species used for modelling AD and frontotemporal dementia (FTD). JNPL3 mice are well-characterized transgenic mice that express human 4R0N tau with a FTDP-17 (P301L) mutation [156]. In particular, the levels of sarkosyl insoluble tau in JNPL3 mice increase in an age-dependent manner and comigrate with insoluble tau from AD and FTDP-17 brains. Thus, treatment with GSK-3 inhibitors should result in a significant reduction of sarkosyl insoluble tau, see, for example, compound **78 **[86, 132]. Besides JNPL3 mice, the transgenic zebrafish larvae have advanced as an AD model system. It combines many of the advantages of invertebrate and vertebrate models. The latest model is a Gal4/UAS-based vector system that efficiently generates transgenic zebrafish overexpressing high levels of human Tau-P301L or other disease-associated proteins. This tau-transgenic fish model could be an effective *in vivo* screening tool to identify quickly promising GSK-3 inhibitors and eliminate compounds without reasonable *in vivo* activity early in the screening process [157, 158]. 

GSK-3 inhibitors have also specific effects on early wildtype zebrafish development when treatment occurs between 4 and 24 hpf. Thus this animal model can be used to test the efficacy of GSK-3 inhibitors *in vivo* [133, 157]. Furthermore, there are two *in vivo *model systems with transiently induced tau hyperphosphorylation that were used to evaluate the activity of GSK-3 inhibitors. One of them is the cold-water stress (CWS) model, which causes a rapid and reversible enhancement of tau phosphorylation in the mouse brain at several GSK-3*β* directed sites such as pSer199, pThr205, pThr231, and pSer396 [125, 159]. The CWS-model proves the significant reduction of tau phosphorylation in **LiCl**- or compound **78**-treated mice [86, 160]. The *in vivo* activities of several other GSK-3 inhibitors (e.g., compound **55** and **96**) were demonstrated in the postnatal rat model [34]. This *in vivo* model takes advantage of the well-characterized GSK-3*β* expression level in the early and later life cycle of rats [161, 162].

## 5. Compounds in Preclinical and Clinical Trials

Currently, several GSK-3 inhibitors pass through preclinical or clinical trials. Subsequently GSK-3 inhibitors are listed with the therapeutic indication of AD. These examples are taken from the database of PharmaProjects by searching for the criteria GSK-3 and Alzheimer (March 2010), the MED-D report of GESENT (May 2009) and ClinicalTrials.gov (August 2011). 

The Wayne State University is currently in phase IV with **lithium** **carbonate** against bipolar disorder whereas the University of Sao Paulo is in phase II with **lithium** **carbonate** against GSK-3 for AD and cognitive impairment. **Lithium** against AD was submitted by the National Institute of Neurological Disorder and Stroke and is in phase II, but there was no update since March 2008. Two compounds from Noscira, **NP-12** (TDZD) and **NP-103**, are in the pre-/clinical trials. **NP-12 **(**NP031112**, **tideglusib**) is currently in a phase IIb clinical trial for AD. At the moment, **NP-103** and **CG-301338** from CrystalGenomics and an GSK-3*β* inhibitor from Takeda are in preclinical trials. The development status of **XD-4241** from Cambrex, **SB-415286** from GlaxoSmith-Kline, a GSK-3*β* inhibitor from Amphora, **SAR-502250** from Sanofi-Aventis, **CEP-16805** from Cephalon, and an GSK-3*β* inhibitor from Lundbeck are not reported. The failure or progress of these compounds into preclinical and clinical trials will stimulate or discourage further research.

## 6. Summary

The moderate inhibition of GSK-3 by selective inhibitors with excellent pharmacokinetic properties and excellent blood-brain barrier permeation holds high potential for the treatment of AD. The failure of the first potent GSK-3 inhibitor by Astra-Zeneca indicates the complexity of this target and the therapeutic window of GSK-3 inhibition in adult mammals. Information on the failure is rather limited, it may be due to potential toxicity of the chemical scaffold: P450-mediated metabolism of thiazoles has resulted in hepatotoxicity and thus failure of phase III candidates previously. 

Biological characterization has advanced GSK-3 as a potential drug target, and the inhibition of this protein kinase by small molecules resulted in significant inhibition of tau phosphorylation. We have described a wide range of molecules that inhibit GSK-3 and discussed their properties. We focused on several inhibitors interacting with the ATP binding pocket of GSK-3*β*. The water molecule interactions are not incorporated in the figures; however, they may play a crucial role in the hydrogen bond network between the inhibitor and the aminoacids. The aminoacids, which are responsible for strong interaction with the enzyme, have been identified. Noteworthy are Asp133, Val135, Glu137, Arg141, Gln185, Asp200, and Arg220, which constitute important aminoacids for interactions with the binding pocket of GSK-3. The conserved salt bridge Lys85/Glu97 materializes as an interesting interaction partner. We summarized crucial biological findings and provided an overview on the *in vivo* effects of some inhibitors. Additional *in vivo* assays can be retrieved via the references denoted in the tables next to the structures. We summarized the GSK-3 inhibitors, which are in pre-/clinical trials with the therapeutic indication of AD. Yet, a review will be biased, and we request your pardon or input, if your favourite inhibitor or *in vivo* studies are not adequately referred to.

## 7. Outlook

GSK-3 is an intriguing enzyme, which plays important roles in the pathogenesis of several diseases, for example, diabetes, cancer, and AD. The literature is immense and quite often provides conflicting statements and observations for this kinase. For example, a few studies observed that Pin1 (peptidyl-prolyl cis-trans isomerase) knockout mice display tau hyperphosphorylation and that this enzyme might have an inhibitive role in phosphorylating tau and GSK-3*β*, thus protecting against AD [163]. Whereas another study ascertained that the GSK-3 inhibitor **BIO** (compound **39**) may be useful in regenerative medicine, by reversibly maintaining human embryonic stem cells in an undifferentiated state [164]. A plethora of GSK-3 inhibitors has been published since it has been linked to AD. Naturally, the vast majority thereof was reported by pharmaceutical companies. Many potent inhibitors with good selectivity have been disclosed so far. The challenge of medicinal chemists will be to develop inhibitors, which translate their potent enzymatic inhibition into cellular settings and finally humans, who will tolerate a moderate GSK-3 inhibition only. A mild GSK-3 inhibition (~35%) is needed because such an inhibition level provides sufficient insulin sensitization without elevation of *β*-catenin levels. Thus a moderate inhibition minimizes significant mechanism-based toxicities, ranging from hypoglycemia to tumorigenesis [165, 166]. A further indication of a moderate GSK-3 inhibition is the application of lithium for the treatment of bipolar disorder. The GSK-3 inhibitor lithium is estimated to inhibit approximately 25% of total GSK-3 activity. It was used for the treatment of bipolar disorder since the 1950s without association of hypoglycemia, increased levels of tumorigenesis, or deaths from cancer [167]. Nevertheless, the established *in vivo *studies have to be accompanied by the investigation of pleiotropic activity and the determination of a safe therapeutic window for chronic GSK-3 inhibition in humans. The X-ray analysis of cocrystallized structures revealed how the inhibitors interact with the ATP-binding pocket and provide information about essential interaction partners to improve potency and selectivity. The comparison of the IC_50_ values will be much easier, if only one stringent GSK-3 *in vitro* assay would be utilized, which employs a defined final ATP and inhibitor concentration as well as a standardized incubation time. Currently, Alon et al. determined that GSK-3*β* is responsible for the phosphorylation of the embryonic tau isoforms in birds, which harbors the GSK-3*β* gene only. In consideration of their and former studies, Alon et al. assume that GSK-3*α* and *β* have distinct roles in phosphorylating tau in adult and embryonic brain in nonvertebrates. Furthermore, they raise the hypothesis that specific inhibition of GSK-3*α* may be useful for therapeutic intervention in AD [168]. This supposition is enhanced by former siRNA experiments [169]. But all GSK-3 inhibitors developed until now inhibit the two isoforms of GSK-3 equipotently, except **Λ-OS1** (**36**) (~7 fold more selective for GSK-3*α*). Still most of the GSK-3 inhibitors fail in model animals despite of their good inhibitory activity in cell free assays. This is frequently due to the lack of selectivity, insufficient cell permeation, and poor blood-brain barrier permeability. Appropriate animal models were developed in transparent zebrafish to study preclinical efficacy, metabolic stability and toxicity, but their potential is not fully exploited. These *in vivo* tests are fast (3 days), relatively inexpensive and suitable for larger screening efforts in 96-well format. Furthermore, it has been observed that resistance arises during the therapy. The problem is that most of the kinase inhibitors are ATP competitive type I inhibitors. A new generation of type II inhibitors, which binds to the ATP site and extends into an allosteric site, may provide a solution [15, 170, 171]. Such novel type II inhibitors must be active, selective and permeate the human blood-brain barrier, which bears further limitations for drug development. However, several pharmaceutical companies continue to develop ATP competitive GSK-3 inhibitors [172, 173]. Just a few pharmaceutical companies have started pre-/clinical trials addressing the druggability of GSK-3 inhibition. Noscira launched the phase IIb trial ARGO of **Tideglusib** (**NP-12**) to treat mild-to-moderate AD patients in April 2011. The trial period will be 65 weeks with 2 dosage regimes of **Tideglusib** (500 and 1000 mg/day oral suspension, ClinicalTrials.gov identifier: NCT01350362). The ongoing clinical trials may lead to a paradigm shift, if the GSK-3 inhibitors display efficacy and safety. 

## Figures and Tables

**Table 1:** Examples of maleimide inhibitors with biological activity against GSK-3, selectivity, X-ray and reference.

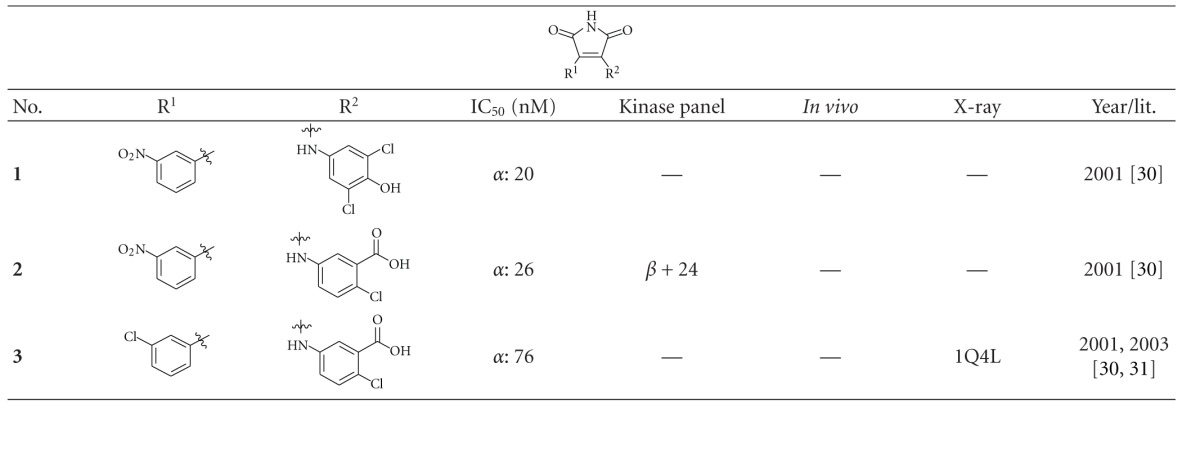

*α*: GSK-3*α*; *β*: GSK-3*β*.

**Table 2:** Examples of indolyl-maleimide inhibitors with biological activity against GSK-3, selectivity, X-ray, and reference.

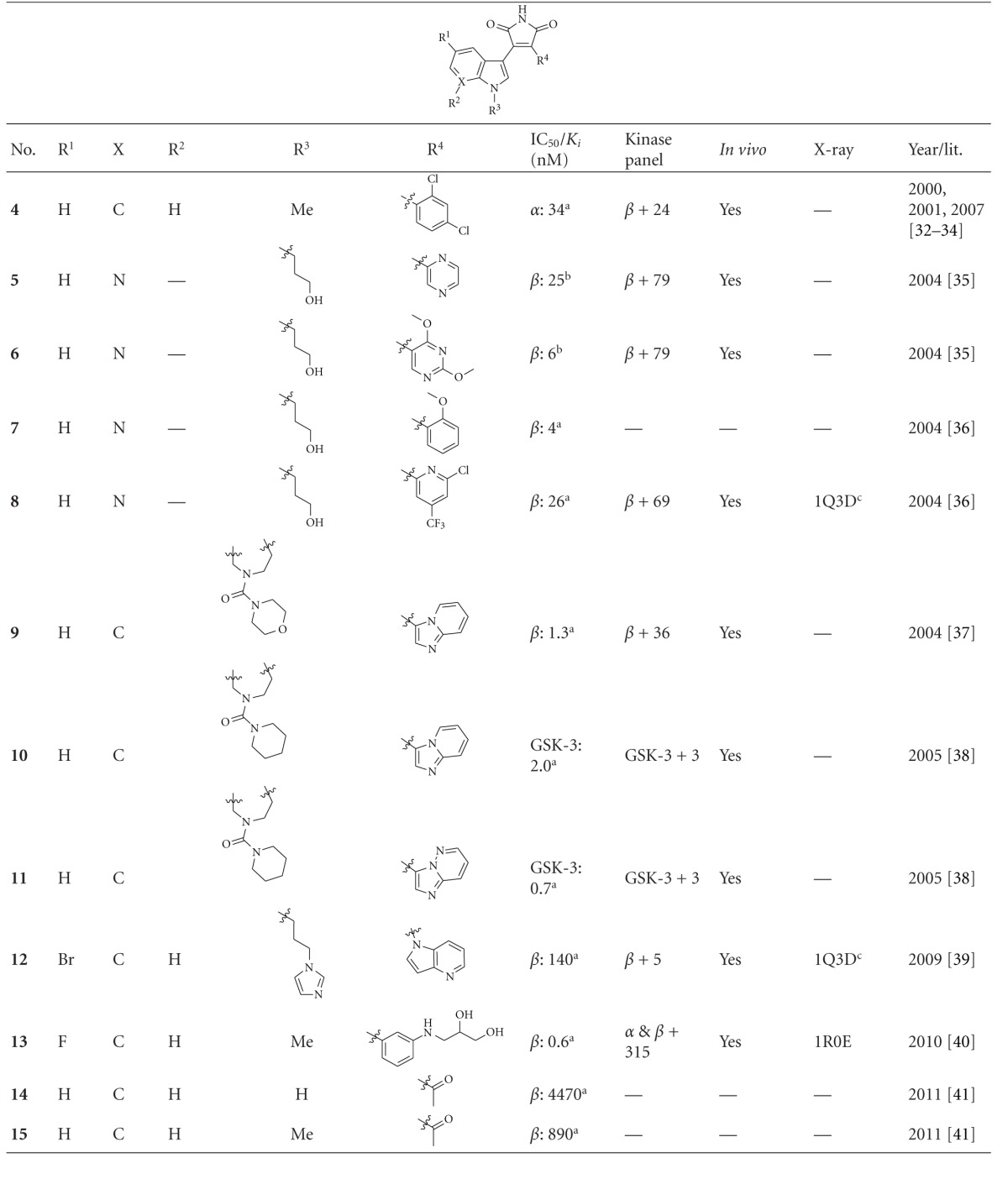

^a^IC_50_ value; ^b^ *K*_*i*_ value; ^c^docking studies, PDB code; *α*: GSK-3*α*; *β*: GSK-3*β*.

**Table 3:** Examples of bisindolyl maleimide and benzofuranyl-indolyl maleimide inhibitors with biological activity against GSK-3, selectivity, X-ray and reference.

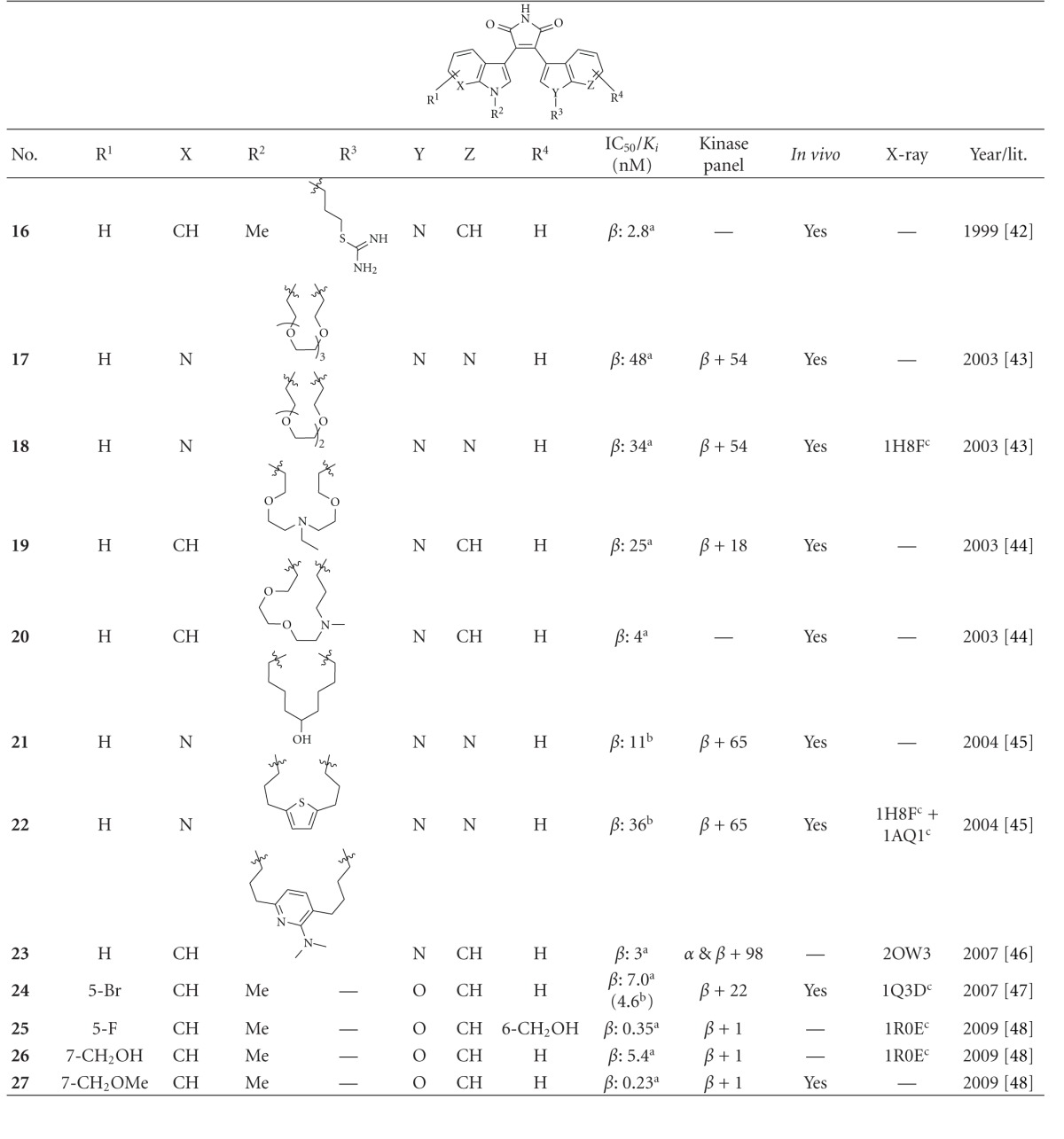

^a^ IC_50_ value; ^b^ *K*_*i*_ value; ^c^Docking studies, PDB code; *α*: GSK-3*α*; *β*: GSK-3*β*.

**Table 4:** Examples of maleimide inhibitors with biological activity against GSK-3, selectivity, X-ray and reference.

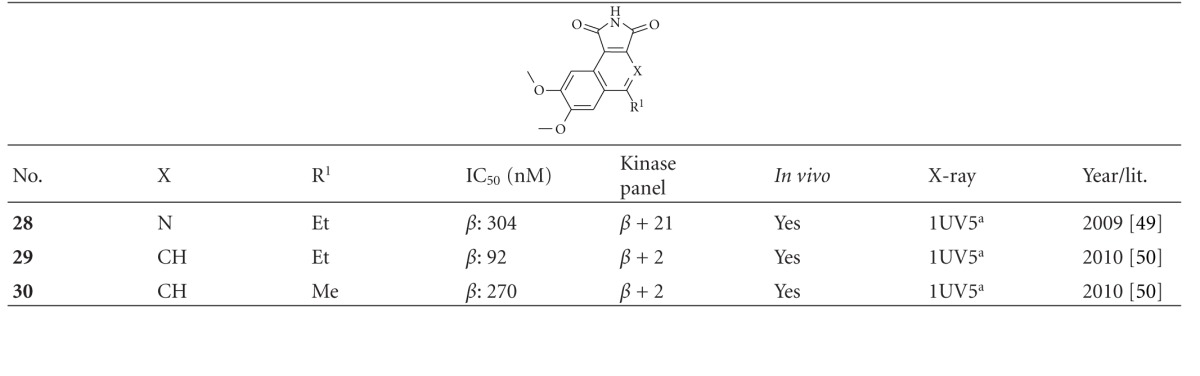

^a^Docking studies, PDB code; *β*: GSK-3*β*.

**Table 5:** Staurosporine and organometallic inhibitors with biological activity against GSK-3, selectivity, X-ray and reference.

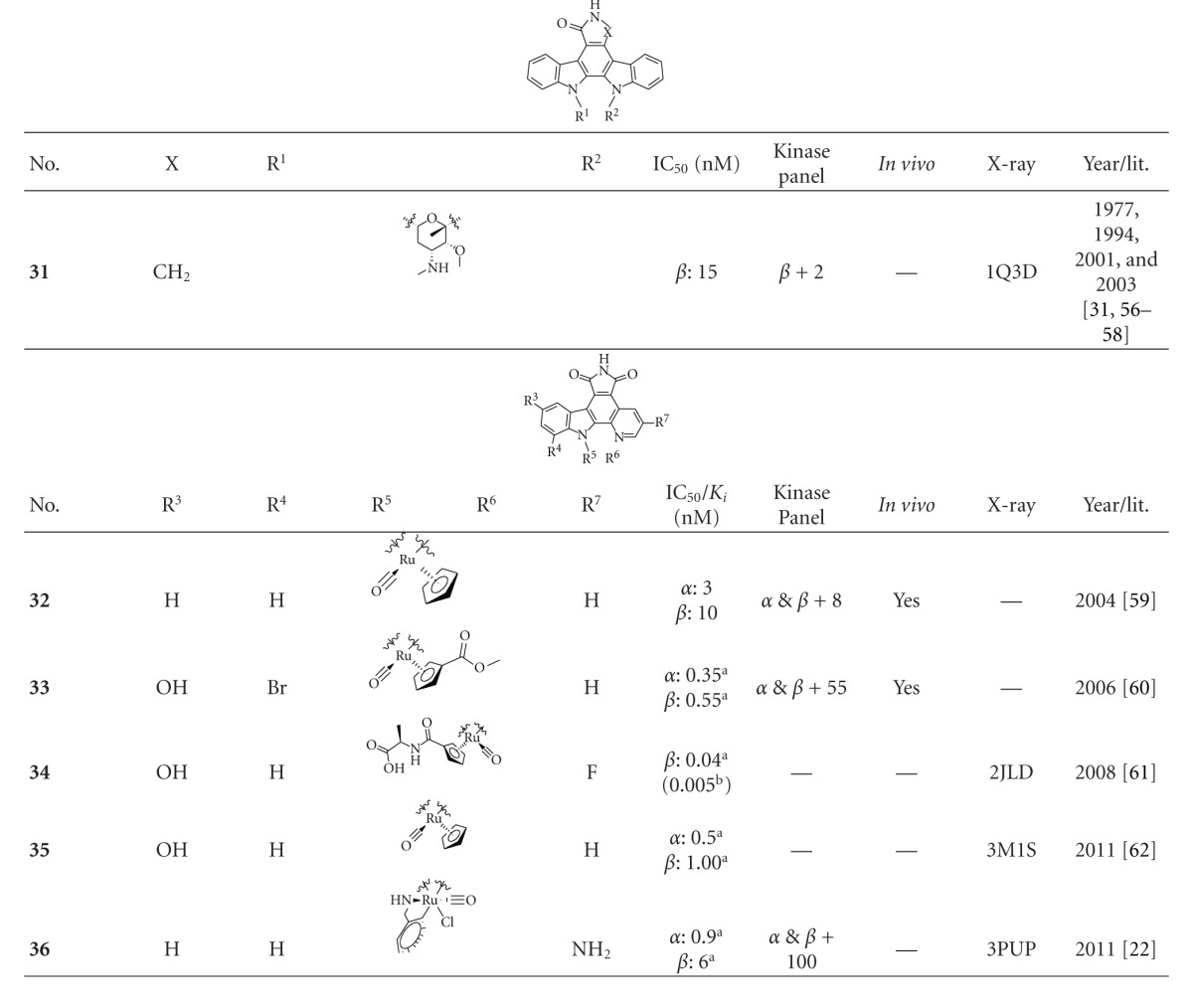

^a^ IC_50_ value; ^b^ *K*_*i*_ value; *α*: GSK-3*α*; *β*: GSK-3*β*.

**Table 6:** Examples of indirubine inhibitors with biological activity against GSK-3, selectivity, X-ray, and reference.

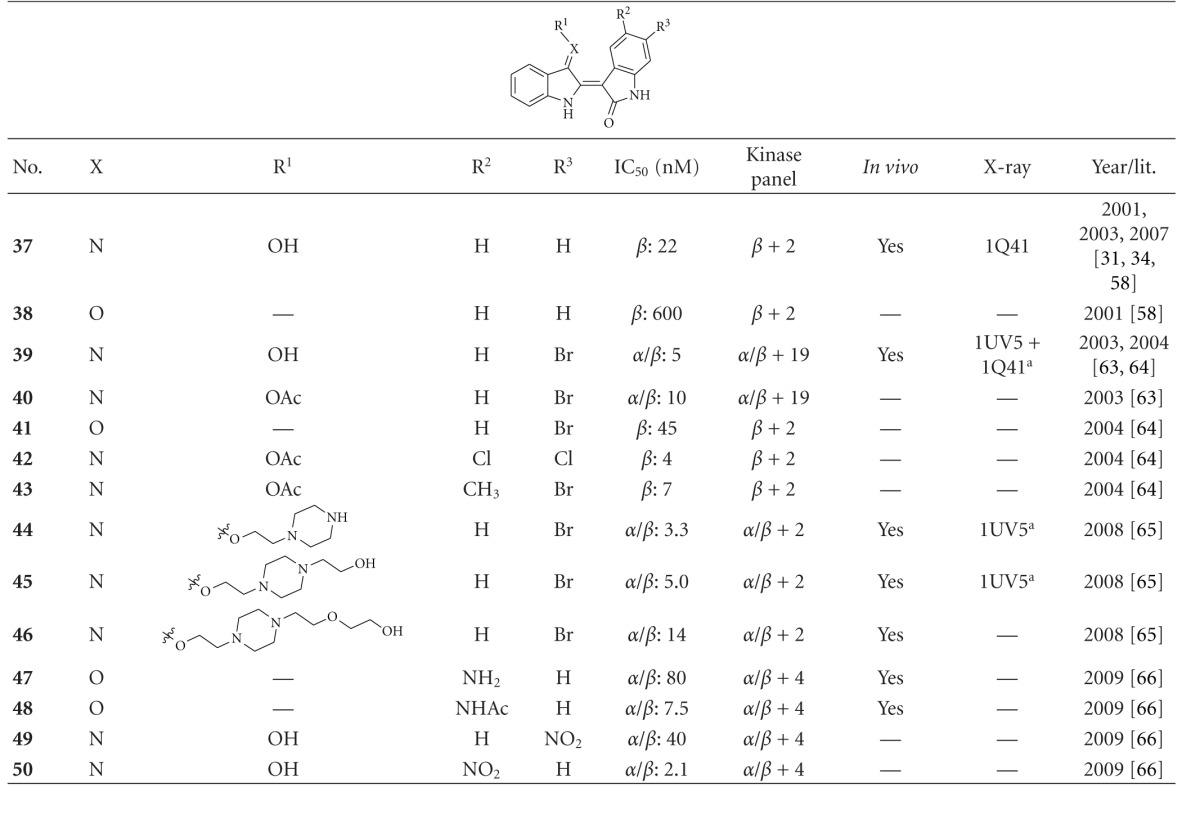

^a^ Docking studies; PDB code; *β*: GSK-3*β*; *α*/*β*: GSK-3*α*/*β*.

**Table 7:** Examples of paullone inhibitors with biological activity against GSK-3, selectivity, X-ray, and reference.

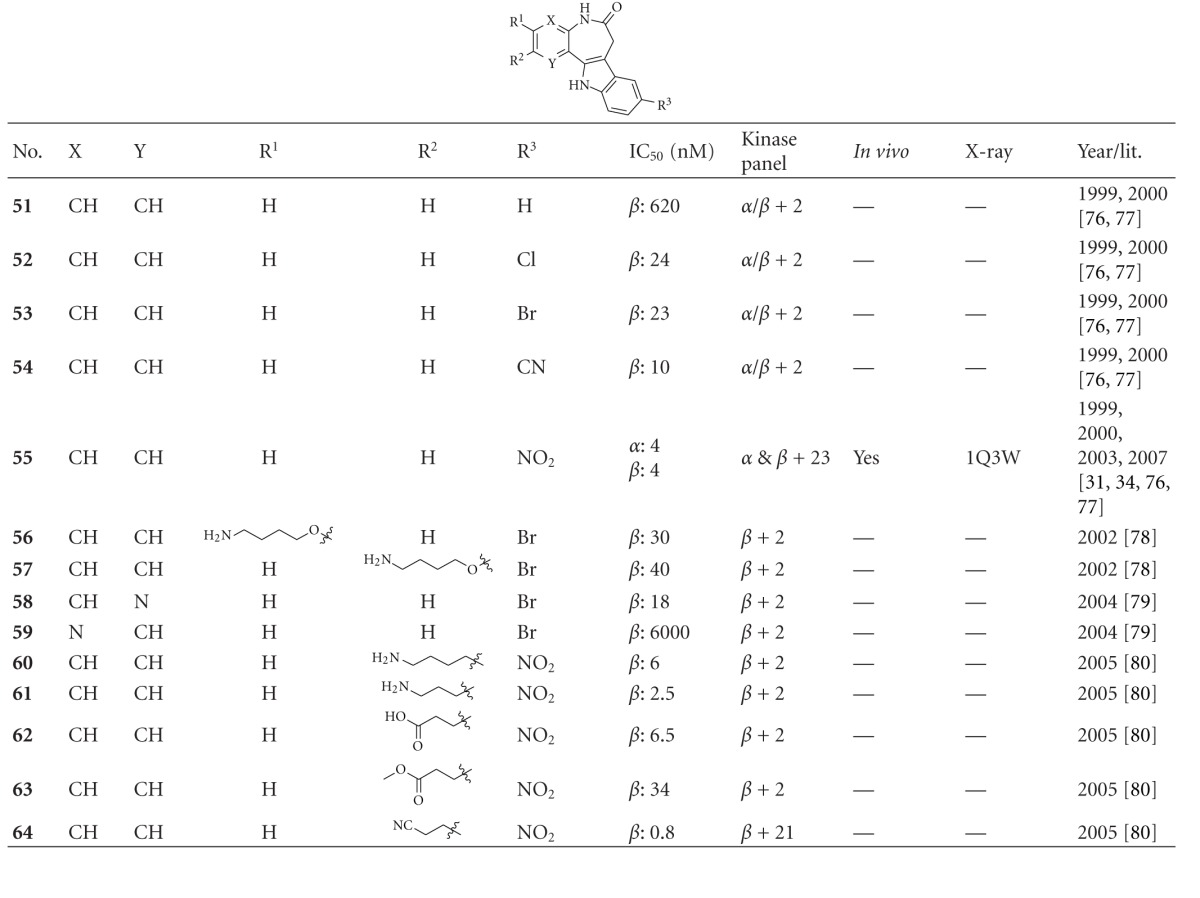

*α*: GSK-3*α*; *β*: GSK-3*β*.

**Table 8:** Examples of pyrazolamide inhibitors with biological activity against GSK-3, selectivity, X-ray, and reference.

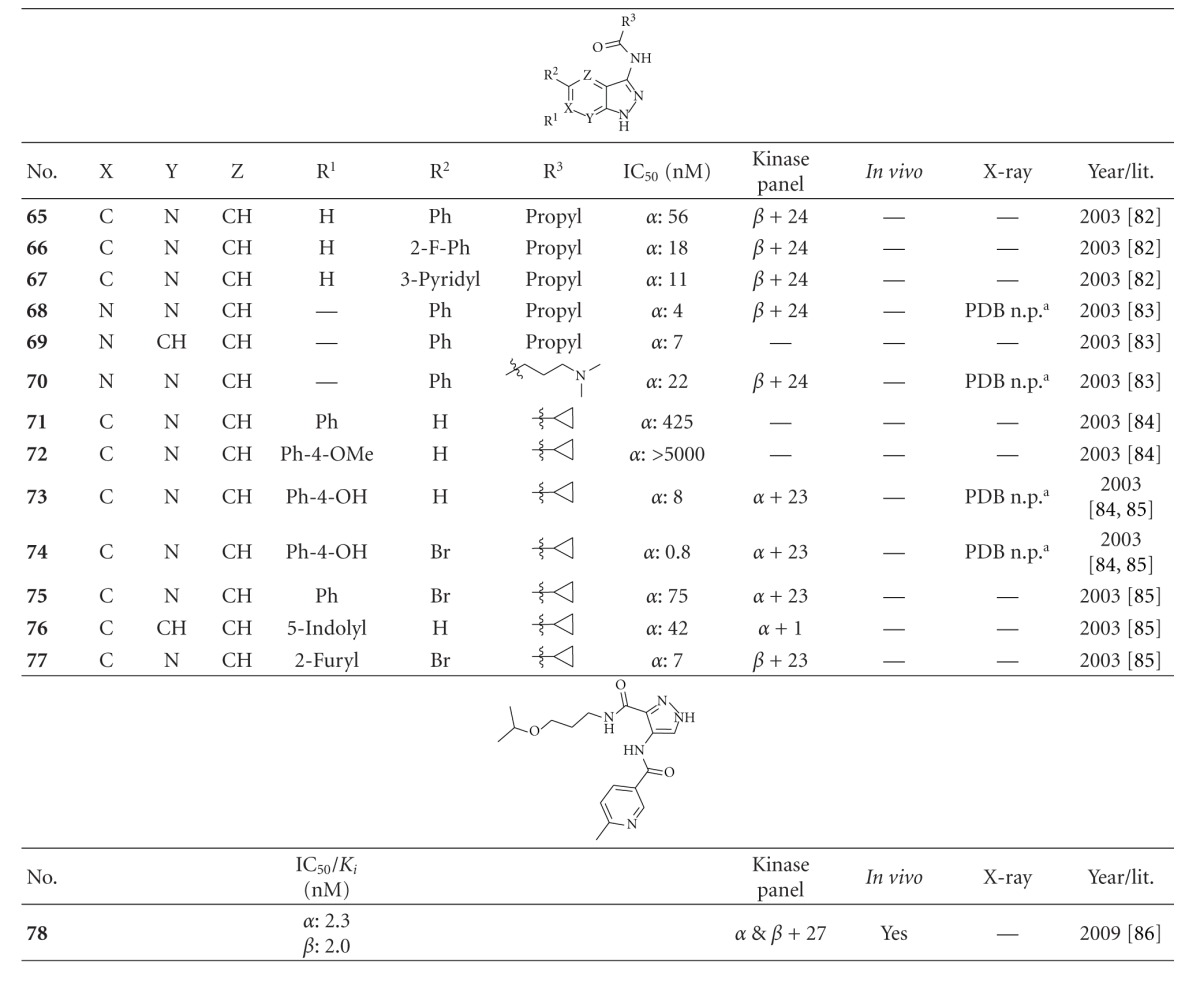

^a^Not published as PDB; *α*: GSK-3*α*; *β*: GSK-3*β*.

**Table 9:** Examples of pyrimidine inhibitors with biological activity against GSK-3, selectivity, X-ray, and reference.

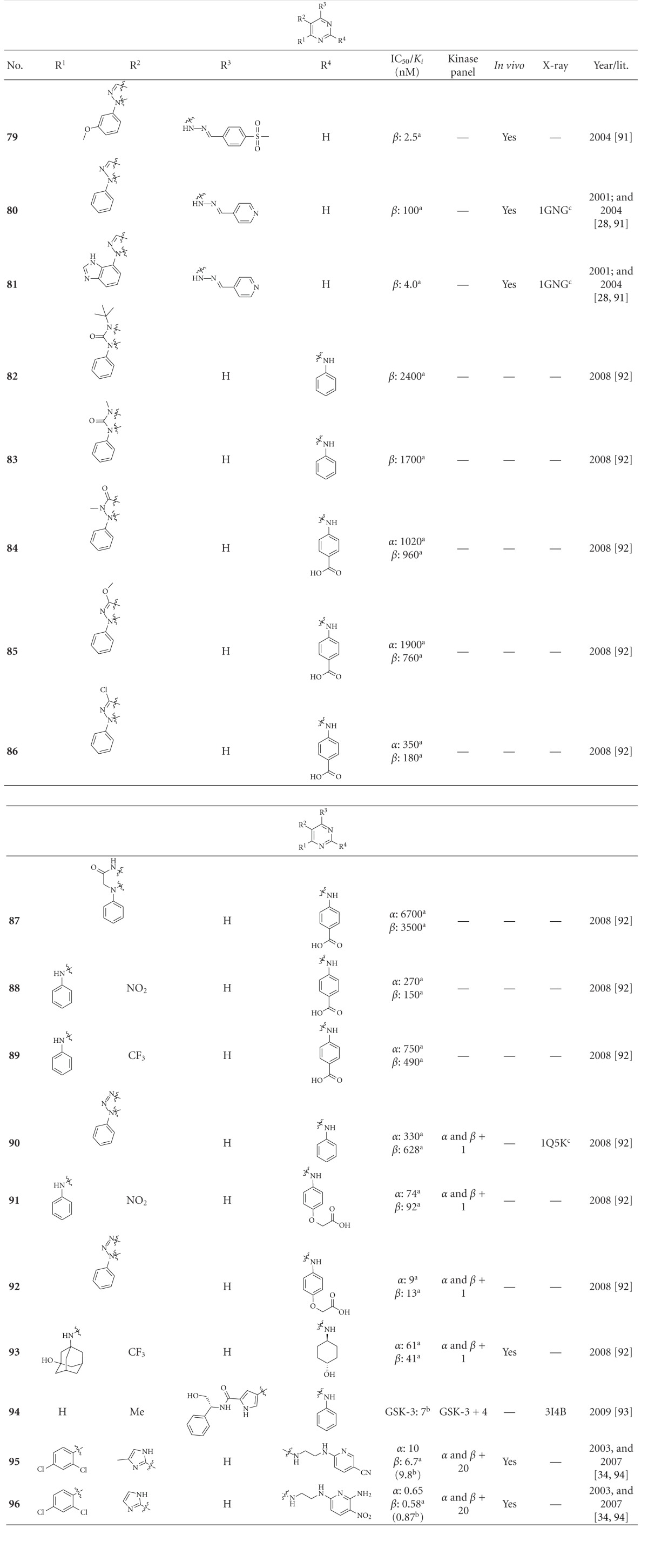

^a^ IC_50_ value; ^b^ *K*_*i*_ value; ^c^docking studies, PDB code (n.d.: not denoted); *α*: GSK-3*α*; *β*: GSK-3*β*.

**Table 10:** Examples of furopyrimidine inhibitors with biological activity against GSK-3, selectivity, X-ray, and reference.

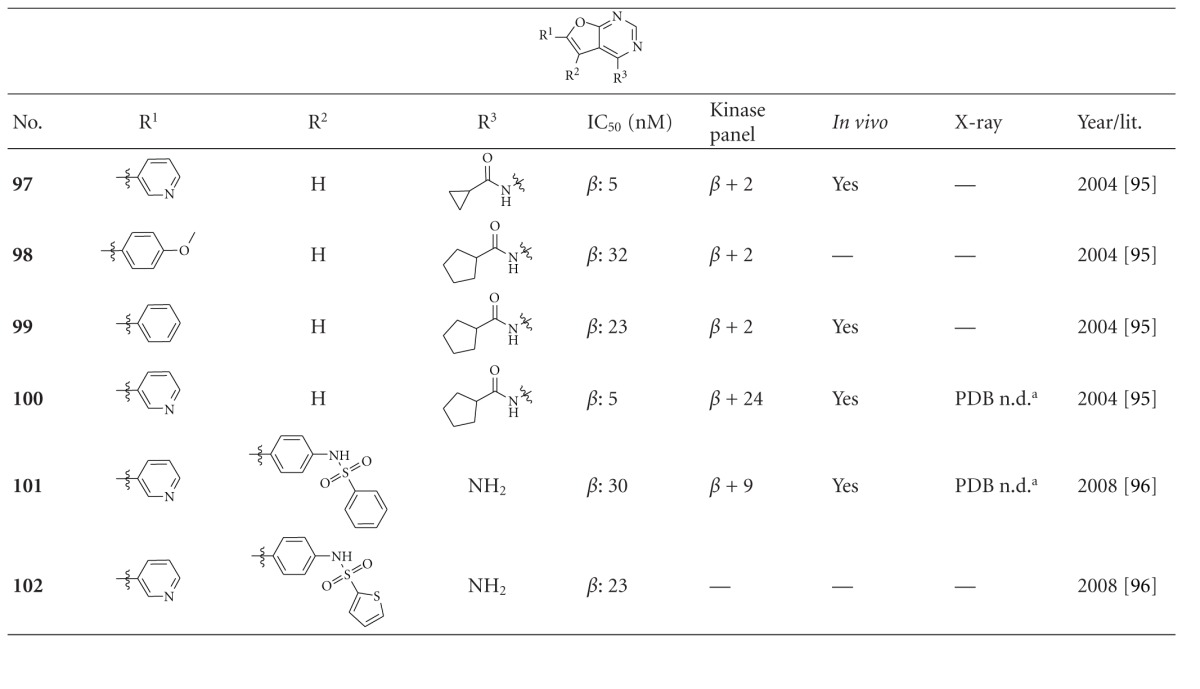

^a^Docking studies, PDB code (n.d.: not denoted); *β*: GSK-3*β*.

**Table 11:** Examples of 1,2,5-oxadiazole inhibitors with biological activity against GSK-3, selectivity, X-ray, and reference.

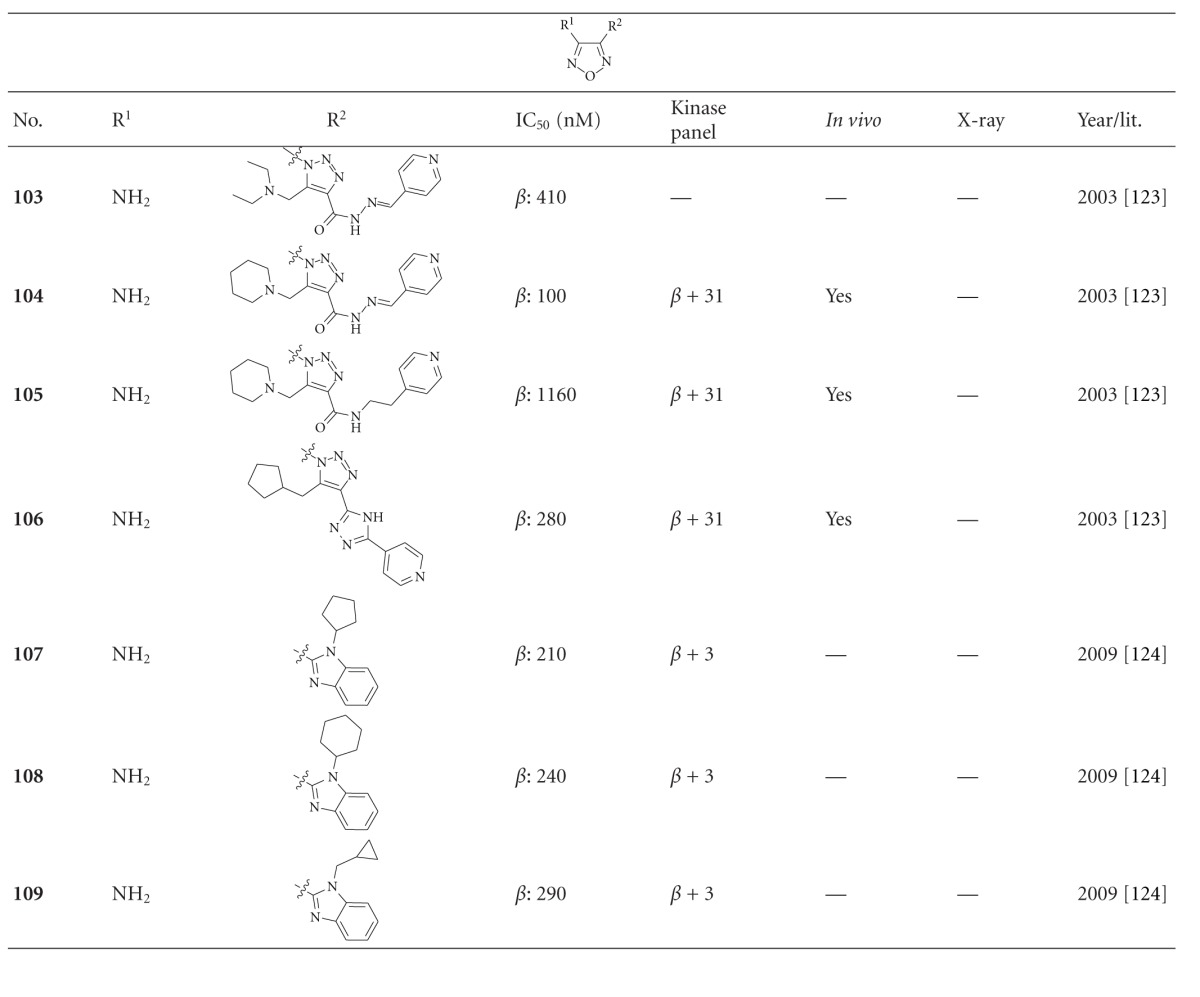

*β*: GSK-3*β*.

**Table 12:** Examples of 1,3,4-oxadiazole inhibitors with biological activity against GSK-3, selectivity, X-ray, and reference.

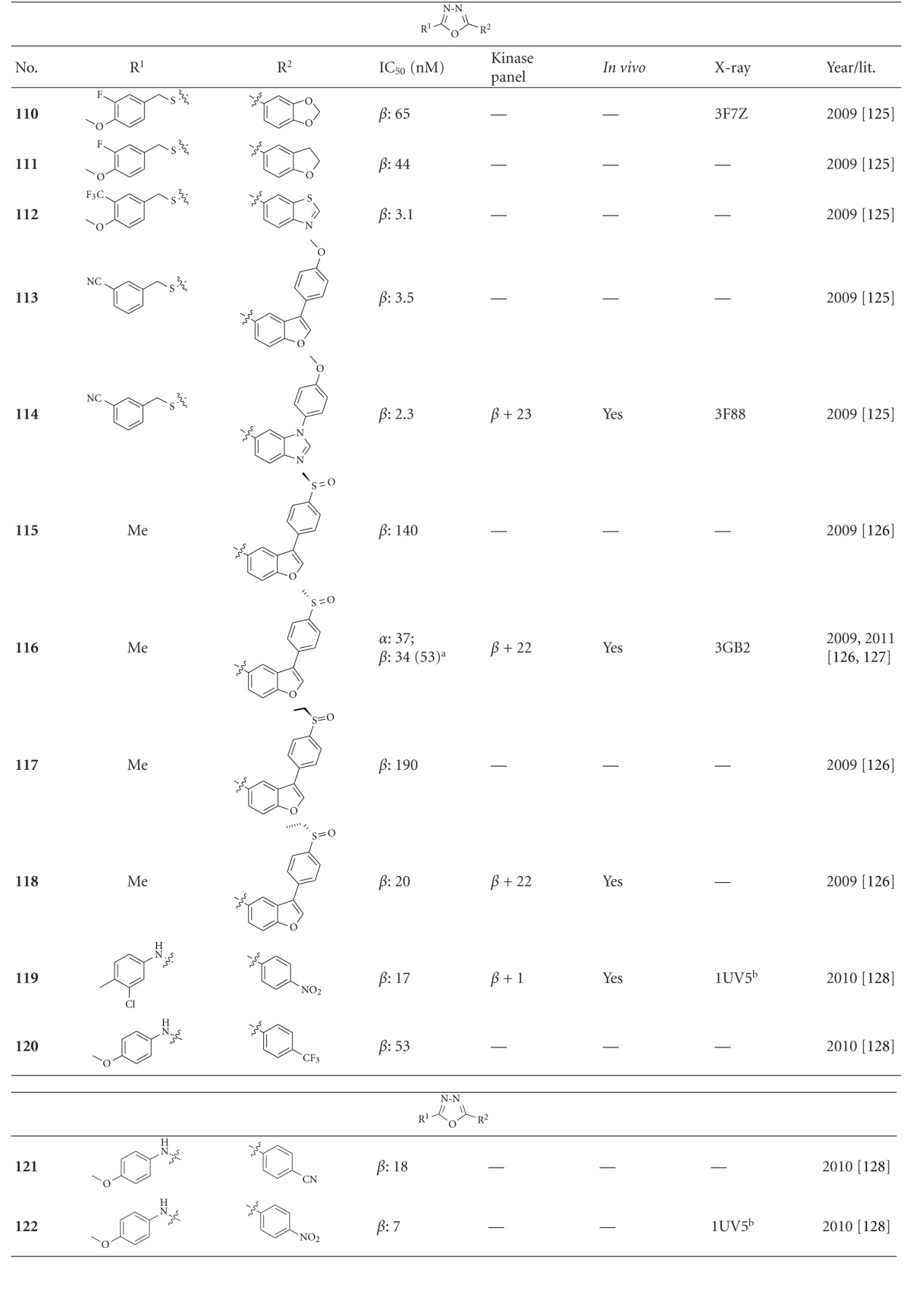

^a^Results of different publications; ^b^docking studies, PDB code (n.d.: not denoted); *β*: GSK-3*β*.

**Table 13:** Examples of 1,2,4-oxadiazole inhibitors with biological activity against GSK-3, selectivity, X-ray, and reference.

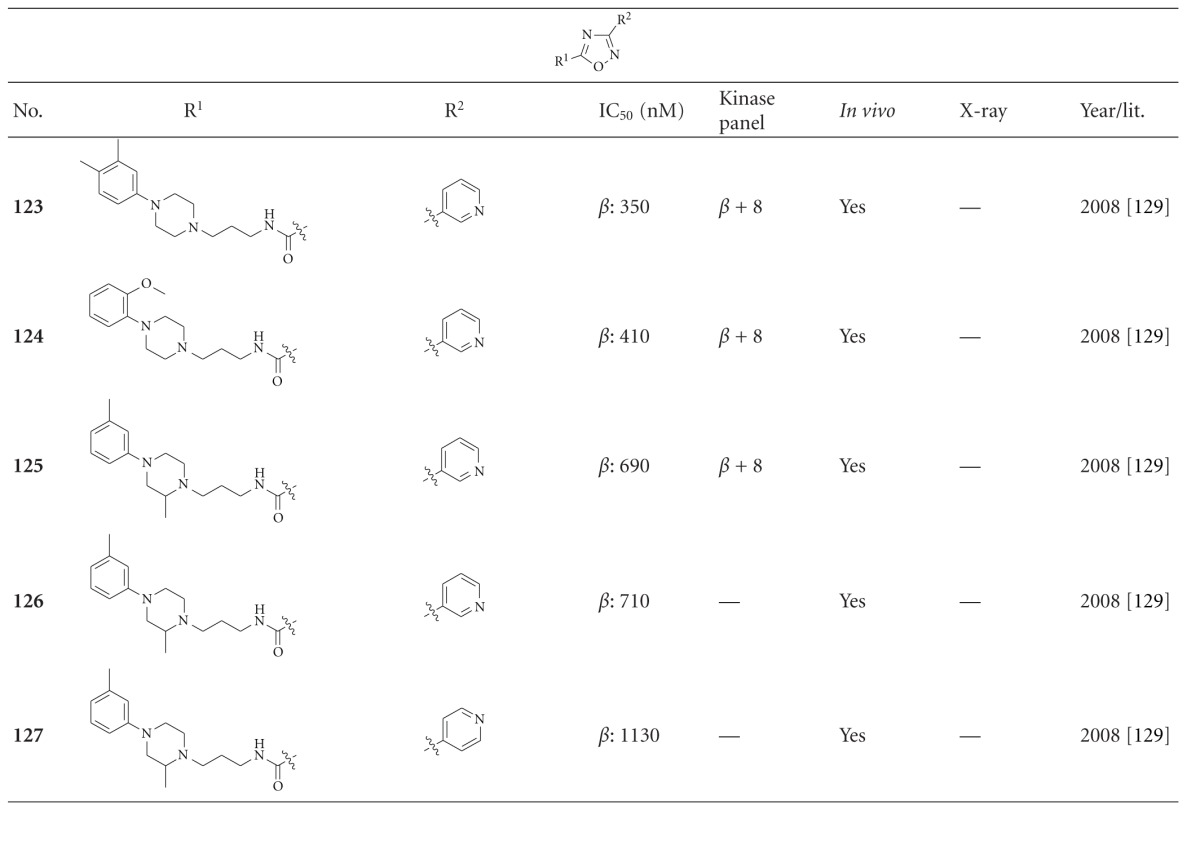

*β*: GSK-3*β*.

**Table 14:** Examples of thiazole inhibitors with biological activity against GSK-3, selectivity, X-ray, and reference.

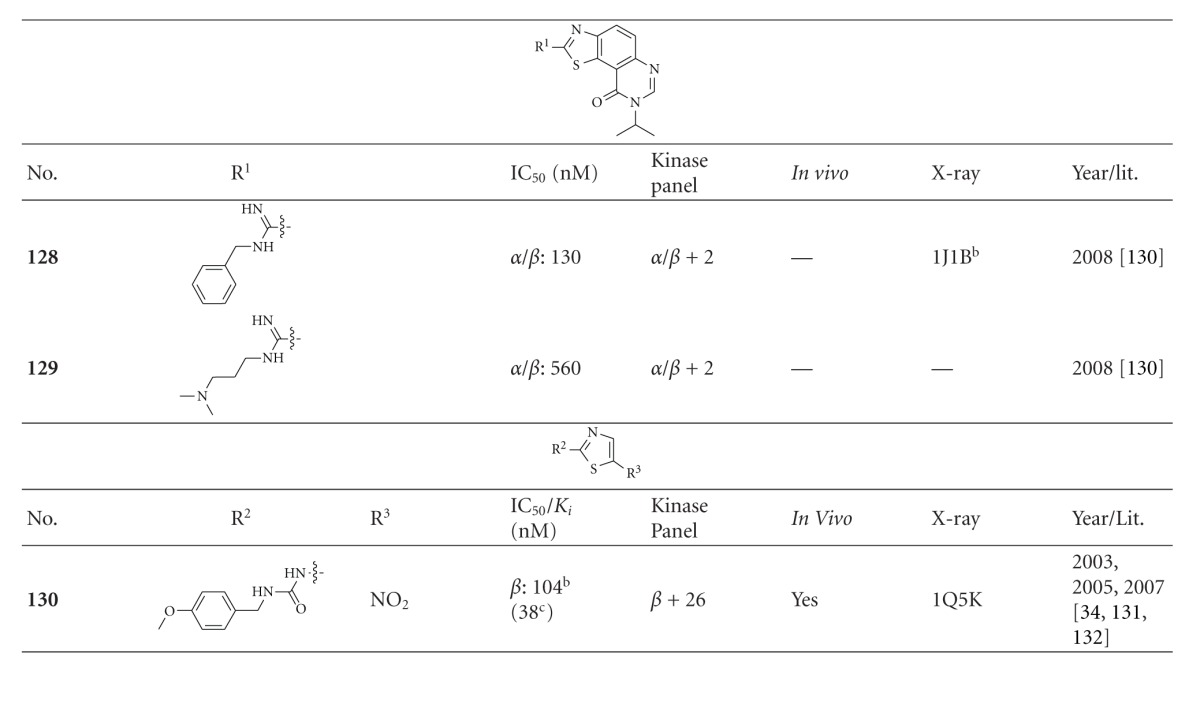

^a^Docking studies, PDB code; ^b^IC_50_ value; ^c^ *K*_*i*_ value; *α*/*β*: GSK-3*α*/*β*; *β*: GSK-3*β*.

**Table:** (a)

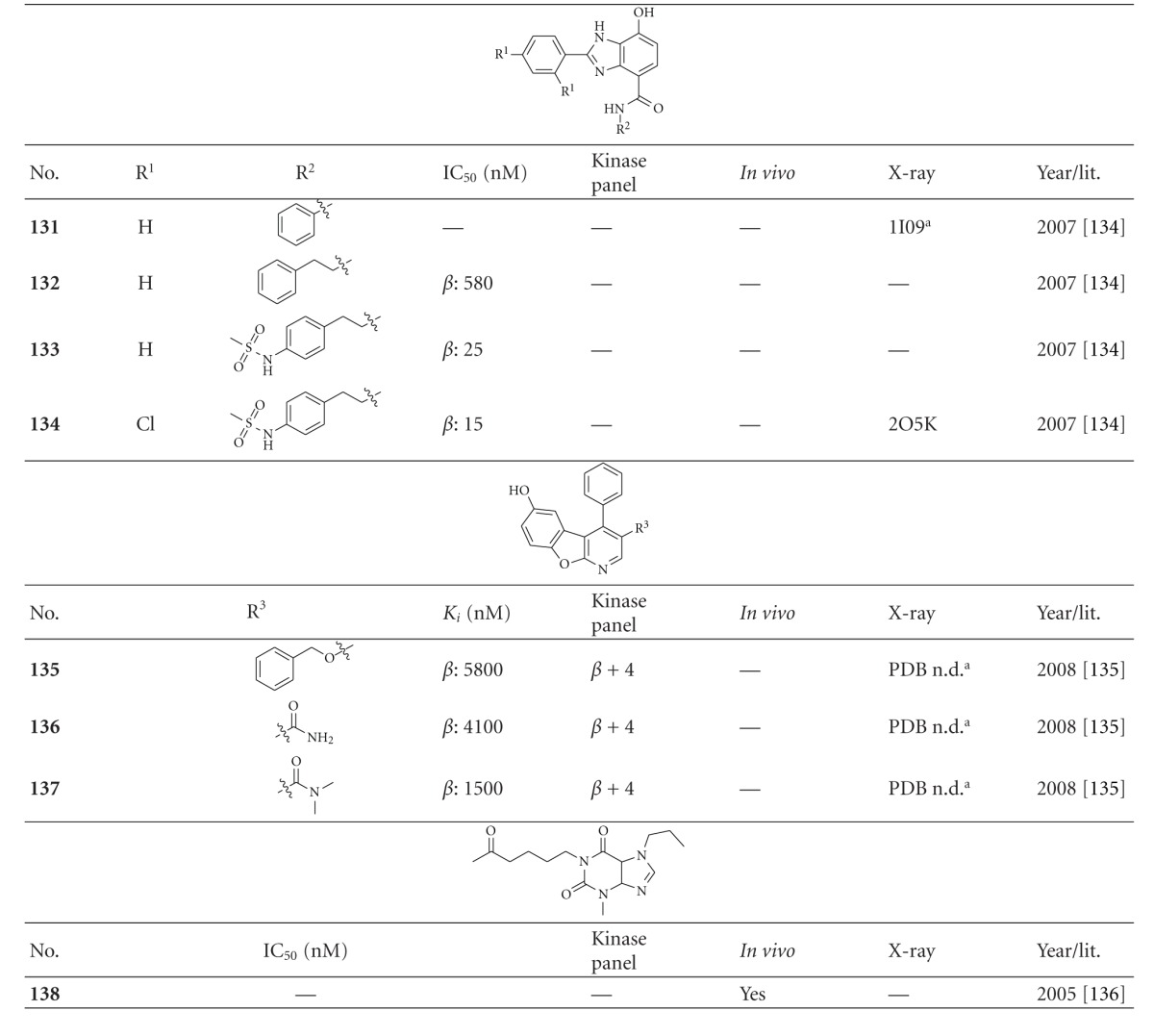

^a^Docking studies, PDB code (n.d.: not denoted); *β*: GSK-3*β*.

**Table:** (b)

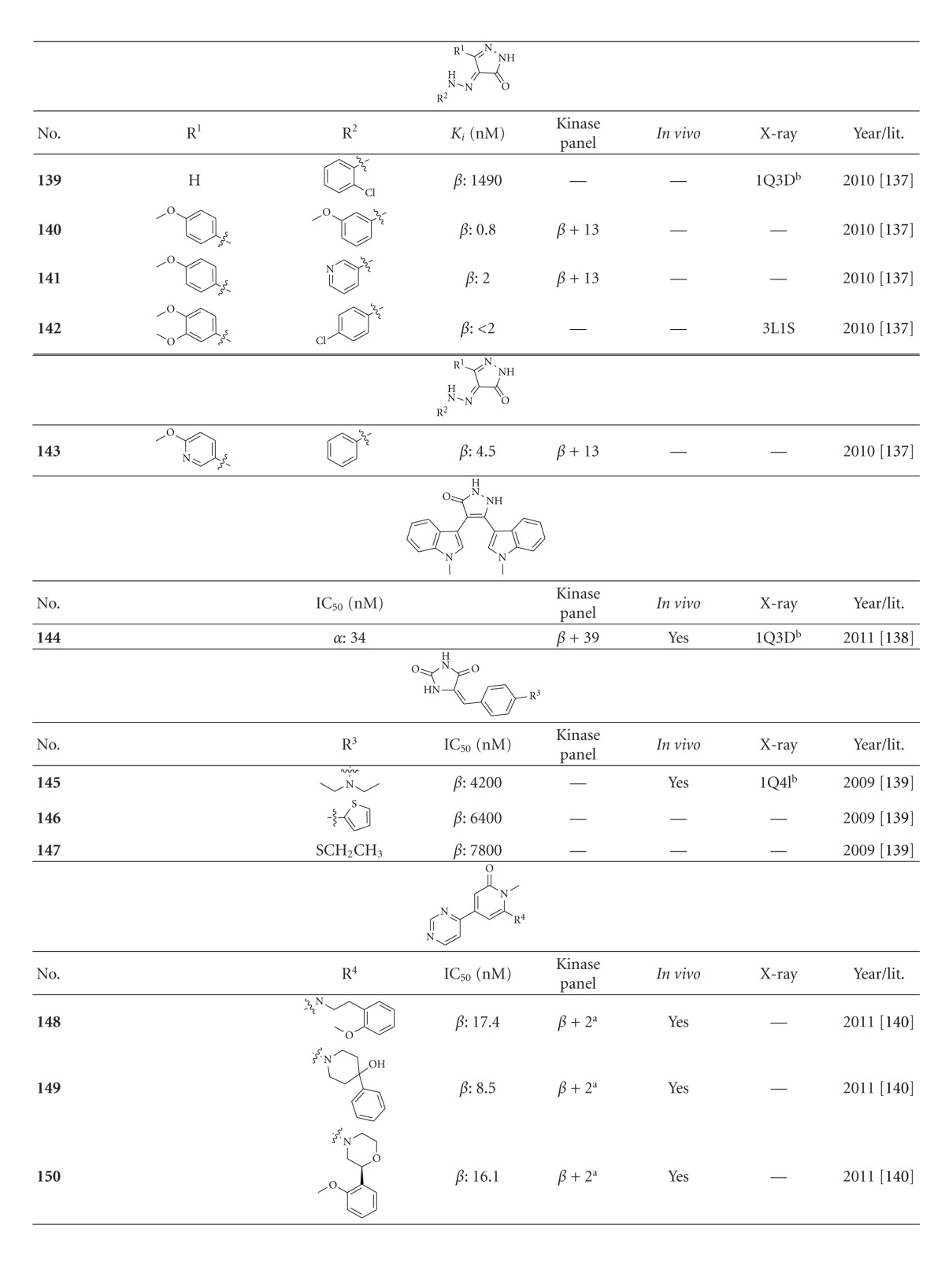

^a^Further, it was noted that the compound was screened against a broad panel of kinases; ^b^docking studies, PDB code; *β*: GSK-3*β*.

**Table:** (c)

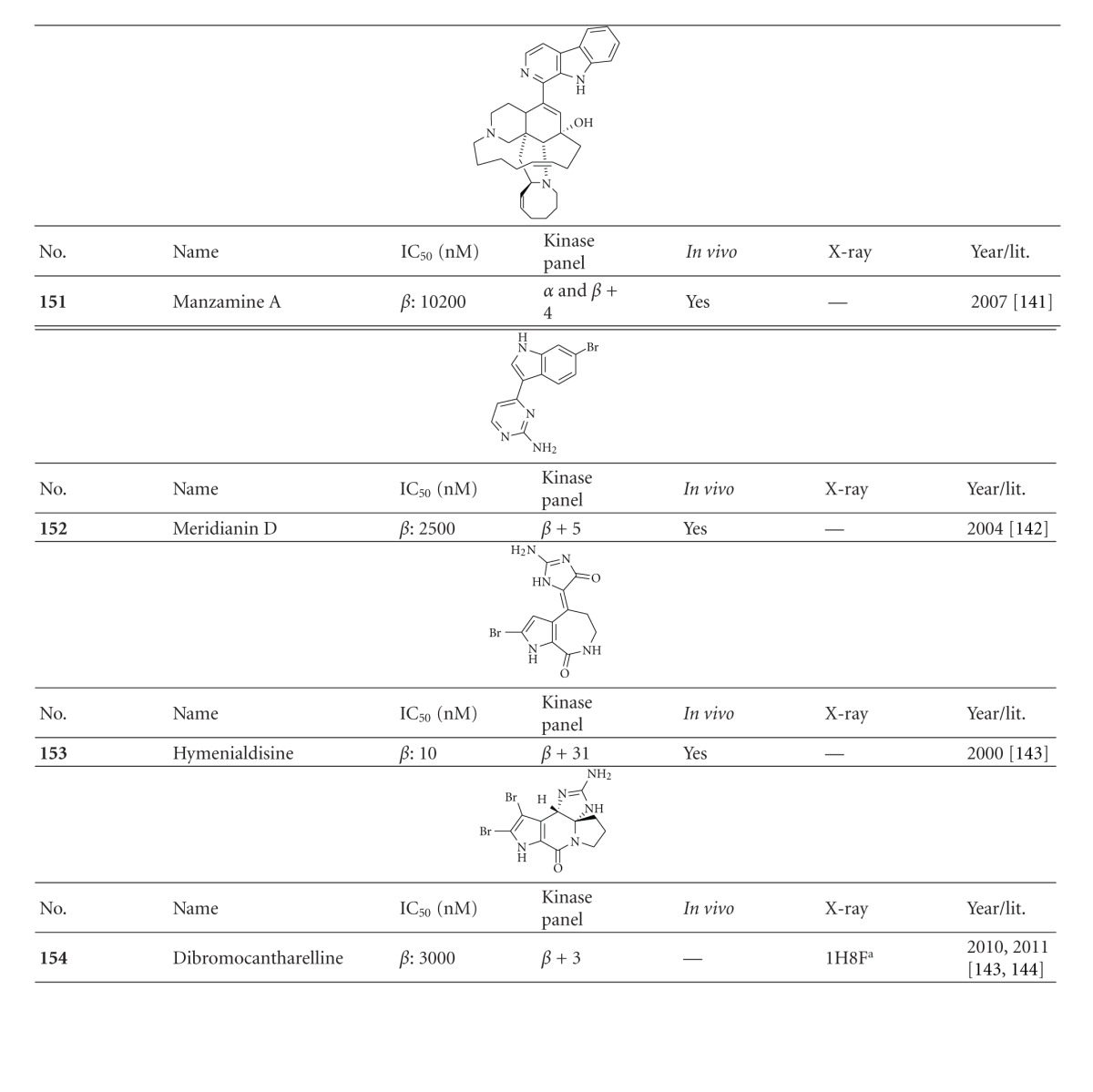

^a^Docking studies, PDB code; *β*: GSK-3*β*.

## Abbreviations

Å: Angstrom
AD: Alzheimer's disease
ApoE: Apolipoprotein E
APP: Amyloid precursor protein
Arg: Arginine
Asp: Aspartic acid
ATP: Adenosine triphosphate
A**β**: **β**-Amyloid
BBB: Blood-brain barrier
CDK: Cyclin-dependent kinase
CK2: Casein kinase 2
CLK1: Dual specificity protein kinase 1
CWS: Cold-water stress
Cys: Cysteine
DMSO: Dimethyl sulfoxide
DYRK1A: Dual specificity tyrosine phosphorylation-regulated kinase 1A
EC50: Half maximal effective concentration
ERK: Extracellular signal-regulated kinase
FAD: Familial Alzheimer's disease
FTD: Frontotemporal dementia
GESENT: Gesellschaft für experimentelle und klinische Neurotherapeutika
Gln: Glutamine
Glu: Glutamic acid
Gly: Glycine
GS: Glycogen synthase
GSK-3: Glycogen synthase kinase 3
h: hour
HMK: Halomethylketone
*i.v.*: Intravenous
IC50: Half maximal inhibitory concentration
Ile: Isoleucine
JNK: c-Jun N-terminal kinase
kDa: kilodalton
KDR: Kinase insert domain receptor
kg: kilogram
*K*_*i*_: Dissociation constant for the inhibitor
Leu: Leucine
LiCl: Lithium chloride
Lys: Lysine
MED-D: Medikamentenentwicklung für Demenzen in Deutschland
mg: milligram
MSK1: Mitogen- and stress-activated protein kinase
MTS: 3-(4,5-dimethylthiazol-2-yl)-5-(3-carboxy-methoxyphenyl)-2-(4-sulfophenyl)-2*H*-tetrazolium
*μ*M: micromolar
NFTs: Neurofibrillary tangles
nM: nanomolar
PDB: Protein Data Bank
Phe: Phenylalanine
PHFs: Paired helical filaments
Pim-1: Proto-oncogene serine/threonine-Protein kinase
Pin1: Peptidyl-prolyl cis-trans isomerase
PKC**β**II: Protein kinase C **β**II
PPF: Propentofylline
Pro: Proline
PS-1/2: Presenilin-1/2
pSer: phosphorylated Serine
pThr: phosphorylated Threonine
RSK1: Ribosomal protein S6 kinase alpha-1, also known as RPS6KA1
*s.c.*: Subcutane
SAD: Sporadic Alzheimer's disease
Ser: Serine
Src: Proto-oncogene tyrosine-protein kinase
TDZD: Thiadiazolidine
Thr: Threonine
Tyr: Tyrosine
Val: Valine
VEGFR: Vascular endothelial growth factor receptor
WHO: World health organization
Wnt: Signaling pathway
X-ray: X-radiation.
